# Phytochemical and Biological Investigation of Two *Diplotaxis* Species Growing in Tunisia: *D. virgata* & *D*. *erucoides*

**DOI:** 10.3390/molecules201018128

**Published:** 2015-10-05

**Authors:** Nizar Ben Salah, Hervé Casabianca, Hichem Ben Jannet, Sophie Chenavas, Corinne Sanglar, Aurélie Fildier, Nabiha Bouzouita

**Affiliations:** 1 Faculté des Sciences de Monastir, Université de Monastir, Avenue de l’Environnement, 5019 Monastir, Tunisia; E-Mail: bensalahnizar9@gmail.com; 2 Laboratoire de Chimie Organique Structurale, Synthèse et Etude Physicochimique–Faculté des Sciences de Tunis, 2092 El Manar, Tunisia; 3 Département Service Central d’analyse, Institut des Sciences Analytiques, Université de Lyon, UMR 5280 CNRS, Université; Lyon 1, ENS-Lyon, 5 Rue de la Doua 69100 Villeurbanne, France; E-Mails: herve.CASABIANCA@isa-lyon.fr (H.C.); s.chenavas@sca.cnrs.fr (S.C.); Corinne.SANGLAR@isa-lyon.fr (C.S.); Aurelie.FILDIER@isa-lyon.fr (A.F.); 4 Laboratoire de Chimie Hétérocyclique, Produits Naturels et Réactivité, Equipe, Chimie Médicinale et Produits Naturels, Faculté des Sciences de Monastir, Université de Monastir, Avenue of the Environment, 5019 Monastir, Tunisia; E-Mail: hich.benjannet@yahoo.fr

**Keywords:** *D*. *erucoides*, *D*. *virgata*, flavonoids, fatty acids, NMR, antioxidant activity, antimicrobial activity

## Abstract

A phytochemical investigation of *D*iplotaxis *virgata* D.C. and *D*. *erucoides* (L.) D.C. (Brassicaceae) offered to the isolation of two new flavonoids isorhamnetin-3-*O*-α-l-glucopyranoside (**1**) and rhamnetin-3,3ʹ-di-*O*-β-d-glucopyranoside (**2**), respectively. Their structures have been elucidated from the extended spectroscopic methods, including 1D- and 2D-NMR, UV and mass spectrometry analysis and by comparison with literature data. The fatty acid composition of the hexane extracts of the two species was also investigated by using GC-MS. The antioxidant activity of ethanol, ethyl acetate, n-butanol extracts and the isolated compounds from the two species was evaluated using DPPH and ABTS^+^ scavenging assays. All the tested samples showed an efficient radical scavenging ability, with IC_50_ values ranging from 16–40 µg/mL for the DPPH and from 17–44 µg/mL for the ABTS^+^ assays. In addition, the antibacterial activity of the prepared extracts and compounds **1** and **2**, determined by well diffusion agar method against two Gram positive and five Gram negative bacteria, was evaluated and the results showed significant effects against all strains used.

## 1. Introduction

Genus *Diplotaxis* (Brassicaceae) is represented by more than 30 species mainly distributed in the north Mediterranean [1]. In Tunisia, beside *Diplotaxis* *virgata* D.C. and *D*. *erucoides* (L.) D.C., five other species can be found [2]. *D. erucoides* and *D*. *virgata* are perennial plants distributed in many sandy and calcareous areas of the Mediterranean basin. In Tunisia, the concentration of distribution of these species is especially high in the South [3].

These plants are economically important; they are used as alternative food and for medical purposes [4,5,6]. In Europe, particularly in Italian and French cuisines, they are consumed directly as salad [7].

Flavonoids are considered members of a class of plant phenolic constituents that have received increasing interest over the last decades. Thousands of them have been isolated and identified, most of which are from food plants. Flavonoids are divided into several subgroups, and their chemical and biological properties can be quite different. Flavonoids are regarded among the main dietary phenolic compounds [8]. That flavonoids possess bioactive effect has been recognized for long, but until recently, data about their bioavailability, metabolic fate, and health effects were limited. It is well known that flavonoids are potent antioxidant metabolites, and therefore one of the main interests in the compounds has involved protection against cardiovascular disease [9].

Lipids, proteins, DNAs and RNAs undergo gradual changes due to the formation of toxic free radicals and auto-oxidation by reactive oxygen species (ROS) into the organisms or the food. Several synthetic antioxidants, such as butylated hydroxyanisole (BHA) and butylated hydroxytoluene (BHT), are currently used as food supplements and stabilizers [10]. However, these synthetic antioxidants have disadvantages due to their possible toxicity and injurious properties to human health [11]. Thus, most consumers prefer additive free foods or a safer approach like the utilization of more effective antioxidant and antimicrobial agents from natural origins. Accordingly, plant extracts and their derived secondary metabolites, such as phenolic components, offer an opportunity in this regard [12].

Flavonoids are present in photosynthetic cells and are usually found in fruits, vegetables, nuts, seeds, stems, flowers, *etc*. This class of compounds has become well known for its antifungal, antiviral and antibacterial activities. However, several studies have examined the structure–antibacterial activity relationship of these compounds. In addition, several research groups have sought to explain the antibacterial mechanisms of action of selected flavonoids. The activity of quercetin, for example, has been at least partially attributed to inhibition of DNA gyrase. It has also been proposed that sophoraflavone G and (−)-epigallocatechin gallate inhibit cytoplasmic membrane function, and that licochalcones A and C inhibit energy metabolism [13]. Available data dealing with phenolic compounds and biological activities of *Diplotaxis* extracts are very scarce.

The aim of the present study was a contribution to the search for new bioactive phenolic compounds through the phytochemical investigation of two Tunisian species, *Diplotaxis virgata* and *D. erucoides,* from which two new flavonoid glycosides have been isolated. The study of the fatty acid composition of the two species has been also carried out. This work was completed by the study of the antioxidant capacity using both DPPH and ABTS^+^ radical scavenging assays, and the antibacterial potential of the n-BuOH extracts and the isolates by the wells diffusion agar method against several human pathogenic bacteria.

## 2. Results and Discussion

### 2.1. Structure Determination

**Figure 1:**
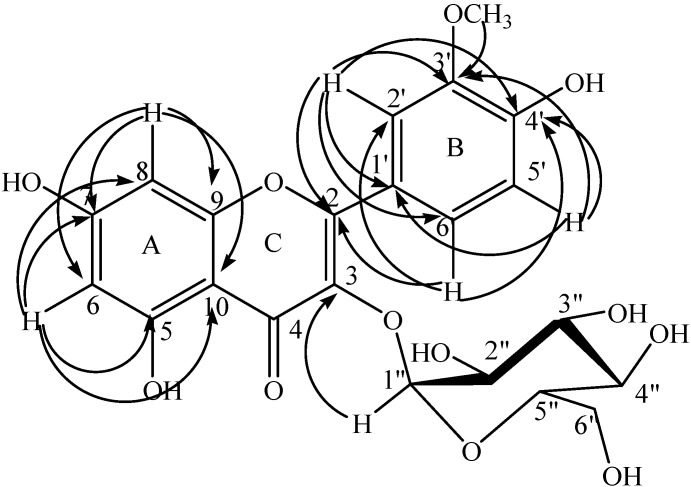
HMBC connectivities of compound **1**.

Compound **1** (Figure 1) was isolated as a pale yellow powder from the *n*-BuOH extract of *D. virgata* flowers. Its UV spectrum exhibited bands at λ_max_ (water:formic acid 99.9v:0.1v/MeOH (50v:50v)): 254 and 340 nm in agreement with a flavonoid skeleton.

The acquisition of the mass spectrum by using the ES-HRMS in a negative mode showed a peak at *m*/*z* 477.1050 ([M − H]^−^) in accordance with the molecular formula of C_22_H_22_O_12_.

The comparison of the spectral data deduced from the ^1^H- and ^13^C-NMR spectra of compound **1** (Table 1) with the literature [14,15,16,17] allowed us to imagine a flavonoid glycoside structure. Its ^1^H-NMR spectrum displayed two singlets at δ_H_ 6.14 and δ_H_ 6.28, attributable to the H-6 and H-8 protons of the 5,7**-**dihydroxylated ring A from the flavonoid skeleton, respectively [14]. Three typical protons appearing at δ_H_ 6.88 ppm (1H, d, *J* = 8.1 Hz, H**-**5ʹ), δ_H_ 7.48 ppm (1H, d, *J* = 7.8 Hz, H**-**6ʹ) and δ_H_ 7.73 ppm (1H, s, H**-**2ʹ) were characteristic to the ring B of the isorhamnetin nucleus [14]. A methoxy group was established by the observation in the ^1^H-NMR spectrum of a singlet at δ_H_ 3.87 ppm (3H, s, OCH_3_) showing a ^1^*J* correlation with its corresponding carbon atom (δ_C_ 57.8) in the HSQC spectrum. The long range correlation H_(OCH3)_/C-3ʹ (δ_C_ 149.0) deduced from the HMBC spectrum was in agreement with the presence of this methoxy group (Figure 1). Its position was supported by the correlations H-2ʹ (δ_C_ 115.0)/C-3ʹ and H-5ʹ (δ_C_ 116.9)/C-3ʹ revealed from the same HMBC spectrum as well as by the *nOe* H-2 (δ_H_ 7.73)/H(OCH_3_) deduced from the NOESY spectrum. The hydroxylation of the ring B at C-4ʹ position was also deduced from the same HMBC spectrum showing the correlations H-2ʹ (δ_H_ 7.73)/C-4ʹ (δ_C_ 151.0), H-5ʹ (δ_H_ 6.88)/C-4ʹ and H-6ʹ (δ_H_ 7.48)/C-4ʹ (Figure 1 and Table 1).

The ^1^H-NMR and the HSQC spectra showing an anomeric signal (δ_H_ 5.17 ppm and δ_C_ 104.4 ppm) together with a set of signals between δ_H_ 3.00 and δ_H_ 5.50 in ^1^H-NMR and δ_C_ 63.0 and 78.7 in ^13^C-NMR revealed the presence of a sugar moiety and thus compound **1** must be a flavonol glycoside. The relatively weak coupling constant (*J* = 4.5 Hz) of the anomeric proton H_1ʹʹ_ at δ_H_ 5.17 implies that the glucose moiety must have an α-glucopyranose form [17]. Additionally, the α-d-glucopyranosyl moiety was assigned on the basis of the observed the ^1^H-^1^H COSY correlations H-1ʹʹ (δ_H_ 5.17)/H-2ʹʹ (δ_H_ 3.48), H-3ʹʹ (δ_H_ 3.48) /H-4ʹʹ (δ_H_ 3.34), H-4ʹʹ /H-5ʹʹ (δ_H_ 3.25) and H-5ʹʹ/H-6ʹʹa,b (δ_H_ 3.54, 3.66) and HSQC experiment together with the HMBC correlation of H-1ʹʹ/C-3 (δ_C_ 136) indicating that the glucopyranosyl system was attached at C-3 (Figure 1 and Table 1).

**Table 1:** ^1^H-NMR (400 MHz) and ^13^C-NMR (100 MHz) data for compound **1** (δ in ppm, in 0.5 mL CD3OD, 0.3 mL D2O).

Position	δ (ppm)		COSY	HMBC	NOESY
	^1^H, mult. (*J* in Hz)	^13^C			
Aglycon					
2		159.5			
3		136			
4		179.9			
5		162.8			
6	6.14 s	100.9	8	10, 8, 7, 5	
7		165.9			
8	6.28 s	96.1	6	10, 9, 7, 6	
9		158.7			
10		106.5			
1ʹ		123.7			
2ʹ	7.73 s	115	6ʹ	1ʹ, 3ʹ, 4ʹ, 2, 6ʹ	3ʹ**-**OMe
3ʹ		149			
4ʹ		151			
5ʹ	6.88 d (*J* = 8.1)	116.9		1ʹ, 3ʹ, 4ʹ	6ʹ
6ʹ	7.48 d (*J* = 7.8)	124.8	6ʹ	4ʹ, 2ʹ, 2	5ʹ
3ʹ**-**OMe	3.87 s	57.8	5ʹ, 2ʹ	3ʹ	2ʹ
Glc					
1ʹʹ	5.17 d (*J* = 4.5)	104.4	2ʹʹ	3	3, 2ʹʹ
2ʹʹ	3.48 m	76.4	1ʹʹ	3ʹʹ	
3ʹʹ	3.48 m	78.2	4ʹʹ	4ʹʹ, 2ʹʹ	
4ʹʹ	3.34 m	71.8	5ʹʹ	3ʹʹ	
5ʹʹ	3.25 m	78.7	6_a_ʹʹ	4ʹʹ	
6_a_ʹʹ	3.54 m	63	5ʹʹ, 6_b_ʹʹ		
6_b_ʹʹ	3.66 m		63	6_a_ʹʹ	

The ^13^C-NMR spectrum revealed the presence of 22 carbon signals among which 16 correspond to the methoxylated flavonol [14] and indicated the presence of an α, β**-**unsaturated ketone at δ_C_ 179.9 (C-4) and nine other quaternary *sp^2^* carbons (Table 1).

Extensive analysis of 1D, 2D-NMR, UV and QTOF mass spectra and by comparing the above NMR data with those of some known flavonoid glycosides [14,15,16,17], compound **1** was deduced to be a flavonol glycoside and accordingly, it was identified as Isorhamnetin**-**3**-***O***-**α**-**l**-**glucopyranoside, a new flavonol glucoside for which we give the trivial name Diplotaxide A (Figure 1).

**Figure 2:**
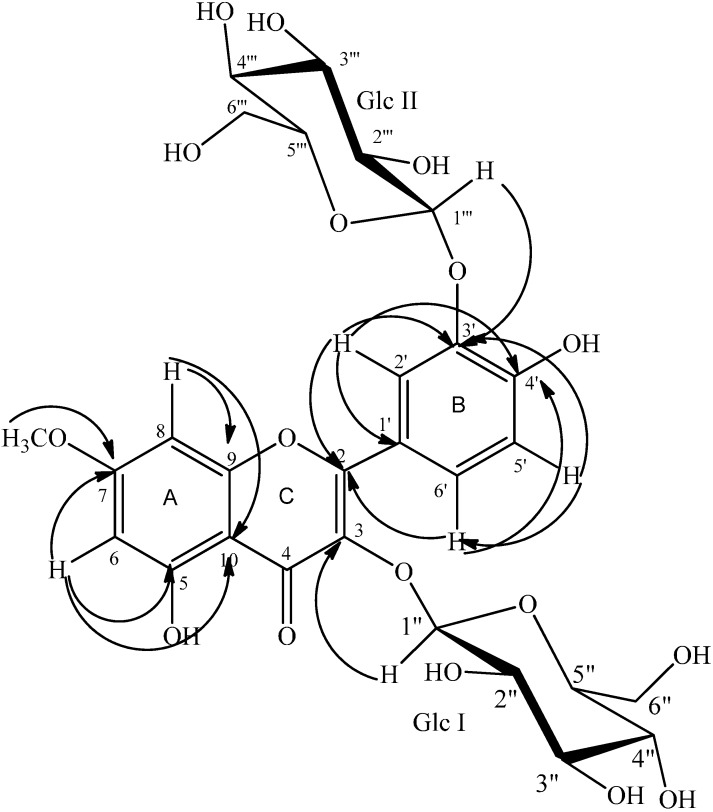
HMBC connectivities of compound **2**.

Compound **2** (Figure 2) was isolated as a pale yellow powder from the *n*-BuOH extract of the non-flowering aerial parts of *Diplotaxis erucoides*. Its UV spectrum exhibited bands at λ_max_ (water:formic acid 99.9v:0.1v/MeOH (50v:50v)): 254 and 340 nm in agreement with a flavonoid skeleton. Its molecular formula was determined as C_28_H_32_O_17_ based on its ESI-HRMS (*m*/*z* 639.1538 [M − H]^−^).

The comparison of the spectral data deduced from the ^1^H- and ^13^C-NMR spectra of compound **2** (Table 2) with the literature [14,15,16,17] allowed us to imagine a flavonoid diglycoside structure.

Its ^1^H-NMR spectrum displayed two proton signals at δ 6.14 ppm (s, H**-**6), and δ 6.28 ppm (s, H**-**8), protons of a 5,7**-**disubstituted ring A from a flavonoid skeleton [14]. Three typical protons appearing at δ_H_ 7.01 ppm (1H, d, *J* = 8.6 Hz, H-5ʹ), δ_H_ 7.81 ppm (1H, dd, *J* = 8.6, 2.1 Hz, H-6ʹ), and δ_H_ 7.99 ppm (1H, d, *J* = 2.1 Hz, H-2ʹ) suggesting the 3ʹ,4ʹ-disubstituted ring B of a flavonoid skeleton.

The same ^1^H-NMR spectrum revealed the presence of a methoxy group by the observation of a singlet at δ_H_ 3.87 ppm (3H, s, OCH_3_) showing a ^1^*J* correlation with its corresponding carbon atom (δ_C_ 58.7) in the HSQC spectrum. The long range correlation H_(OCH3)_/C-7 (δ_C_ 168.2) deduced from the HMBC spectrum was in concordance with the presence of this methoxy group (Figure 2 and Table 2). Its position was supported by the correlations H-6/C-7 and H-8/C-7 deduced from the same HMBC spectrum as well as via the dipolar interaction of the methoxy protons with both H-6 and H-8 in the NOESY spectrum (Table 2).

**Table 2:** ^1^H-NMR (400 MHz) and ^13^C-NMR (100 MHz) data for compound **2** (δ in ppm, in 0.5 mL CD_3_OD, 0.3 mL D_2_O).

Position	δ (ppm)		COSY	HMBC	NOESY
	^1^H, mult. (*J* in Hz)	^13^C			
Aglycon					
2		160			
3		136.7			
4		180.6			
5		163			
6	6.34 s	100.3	8	10, 8, 7, 5	7**-**OMe
7		168.2			
8	6.64 s	94.5	6	10, 9, 7, 6	7**-**OMe
9		159.2			
10		107.5			
1ʹ		124.4			
2ʹ	7.99 d (*J* = 2.1)	121	6ʹ	6ʹ, 3ʹ, 4ʹ, 2	
3ʹ		147.1			
4ʹ		152.3			
5ʹ	7.01 d (*J* = 8.6)	118.2	6ʹ	1ʹ, 4ʹ, 3ʹ	6ʹ
6ʹ	7.81 dd (*J* = 8.6, 2.1)	128	5ʹ, 2ʹ	4ʹ	5ʹ
7**-**OCH_3_	3.87 s	58.7		7	6, 8
(Glc I)					
1ʹʹ	5.11 d (*J* = 7.6)	104.8	2ʹʹ	3	
2ʹʹ	3.48 m	76.4	1ʹʹ		
3ʹʹ	3.43 m	78.5			
4ʹʹ	3.34 m	71.9			
5ʹʹ	3.19 m	79.1	6_a_ʹʹ, 6_b_ʹʹ		
6_a_ʹʹ	3.52 m	63.2	5ʹʹ		
6_b_ʹʹ	3.64 m	63.2	5ʹʹ		
(Glc II)					
1ʹʹʹ	4.95 d (*J* = 7.4)	105.2	2ʹʹʹ	3ʹ	
2ʹʹʹ	3.58 m	78.1	1ʹʹʹ		
3ʹʹʹ	3.47 m	71.9	4ʹʹʹ		
4ʹʹʹ	3.58 m	75.6	5ʹʹʹ		
5ʹʹʹ	3.47 m	79	6_a_ʹʹʹ,6_b_ʹʹʹ		
6_a_ʹʹʹ	3.77 m	63.2	5ʹʹʹ, 6_b_ʹʹʹ		
6_b_ʹʹʹ	3.91 m	63.2	5ʹʹʹ, 6_a_ʹʹʹ		

Additionally, two β-d-glucopyranosyl moieties were assigned on the basis of the observed signals in the region 3.00–5.50 ppm of the ^1^H-NMR spectrum, reinforced by the ^1^H-^1^H COSY correlations H-1ʹʹ (δ_H_ 5.11)/H-2ʹʹ (δ_H_ 3.48), H-3ʹʹ (δ_H_ 3.43) /H-4ʹʹ (δ_H_ 3.34), H-4ʹʹ/H-5ʹʹ (δ_H_ 3.19) and H-5ʹ/H-6ʹa,b (δ_H_ 3.52, 3.64) corresponding to the first glucopyranosyl system and H-1ʹʹʹ (δ_H_ 4.95)/H-2ʹʹʹ (δ_H_ 3.58), H-2ʹʹʹ (δ_H_ 3.43) /H-3ʹʹʹ (δ_H_ 3.47), H-3ʹʹʹ/H-4ʹʹʹ (δ_H_ 3.58), H-4ʹʹʹ/H-5ʹʹʹ (δ_H_ 3.47) and H-5ʹʹʹ/H-6ʹʹʹa,b (δ_H_ 3.77, 3.91) corresponding to the second glucopyranosyl system (Table 2). The above attributions were reinforced by the HSQC experiment showing all ^1^*J* correlations especially those of the anomeric protons with the corresponding carbons H-1ʹʹ/C-1ʹʹ (δ_C_ 104.8) and H-1ʹʹʹ/C-1ʹʹʹ (δ_C_ 105.2) (Table 2) [15].

The ^13^C-NMR spectrum revealed the presence of 28 carbon signals among which 16 correspond to the methoxylated flavonol [14] and indicated the presence of an α,β**-**unsaturated ketone at δ_C_ 180.6 (C-4) and 10 quaternary *sp^2^*carbons. This assignment was supported by the literature data relative to flavonoid glycosides in plants, namely rhamnetin and isorhamnetin derivatives [14,15,16,17].

The position of the first sugar (Glc I) moiety was ascertained by the long range correlation between the anomeric proton H-1ʹʹ and the quaternary carbon C-3 (δ_C_ 136.7) of the ring C of the flavonol moiety observed in the HMBC spectrum. On the other hand, the position of the second glucopyranosyl system (Glc II) was deduced without ambiguity from the same spectrum by the observation of the correlations H-1ʹʹʹ/C-3ʹ (δ_C_ 147.1), H-2ʹ/C-3ʹ and H-5ʹ/C-3ʹ (Figure 2).

Careful analysis of 1D, 2D-NMR, UV and QTOF mass spectra and by comparing the above NMR data with those of some known flavonoid glycosides [14,15,16,17], compound **2** was deduced to be a flavonol diglycoside and, accordingly, it was identified as rhamnetin-3,3ʹ-di-*O*-β-d-glucopyranoside, isolated for the first time, and for which we give the trivial name Diplotaxide B (Figure 2).

### 2.2. Fatty Acid Composition (GC-MS)

Fatty acid composition of the two hexane extracts of *D*. *virgata* flowers and non-flowering aerial parts of *D*. *erucoides* are presented in Table 3. The fatty acid profiles were obtained using gas chromatography and revealed five fatty acids with palmitic acid (18.23%), linolelaidic acid (15.66%) and linoeic acid (23.65%) as predominant compounds in the hexane extract of *D. viragata* (HFF-DV), whereas only three acids were detected in the hexane extract of *D. erucoides* (HFPA-DE). They were identified to be palmitic acid (14.35%), linolelaidic acid (6.91%) and linoleic acid (29.10). In the two hexane extracts, the unsaturated acids constitute the main fraction (42.73% in HFF-DV and 36.01% in HFPA-DE). Moreover, linoleic acid was found to be predominant in the two fractions (Table 3).

**Table 3:** Fatty acid composition of the hexane fractions of *D. viragata* and *D*. *erucoides.*

Fatty Acid	HFF-DV (%)	HFPA-DE (%)
Palmitic acid C16:0	18.23	14.35
Stearic acid C18:0	3.08	-
Oleic acid C18:1	3.42	-
Linoleic acid C18: 2	23.65	29.1
Linolelaidic acid C18: 2	15.66	6.91

HEF-DV: Hexane fraction of *Diplotaxis virgata* flowers, HFPA-DE: Hexane fraction of *Diplotaxis* *erucoides* non**-**flowering aerial parts.

### 2.3. Antioxidant Activity

#### 2.3.1. DPPH Radical Scavenging Activity

The DPPH assay is a preliminary assay to study the antioxidant effect of chemicals. Antioxidants, in interaction with DPPH, either transfer an electron or hydrogen atom to DPPH, thus neutralizing its free radical character [18]. The color changes from purple to yellow and its absorption at the wavelength 517 nm decreases. Free radical scavenging properties of crude ethanol extracts from *D. virgata* and *D. erucoides* and those of their ethyl acetate and n-butanol fractions as well as of the isolates **1** and **2** are reported in Table 4.

According to the results given in Table 4, all extracts, fractions and pure compounds tested showed a strong DPPH radical scavenging activity compared to that of synthetic antioxidant BHT. It ranged from 16.01 ± 0.13 μg/mL to 40.05 ± 1.78 μg/mL (for D. *virgata*) and from 18.00 ± 0.01 μg/mL to 27.02 ± 1.53 μg/mL (for D. *erucoides*). Lower IC_50_ value indicated higher antioxidant activity. In both cases, the n-BuOH fractions exhibited a radical-scavenging activity slightly higher than that of the EtOAc ones. This finding could be explained by their richness in phenolic metabolites as compounds **1** (16.01 ± 0.13 μg/mL) and **2** (18.00 ± 0.01 μg/mL) which exhibited practically the same activity as that of BHT (18.00 ± 0.23 μg/mL). The antioxidant activity of our extracts was found comparable to that of extracts from *Diplotaxis harra* and *D. simplex* [19]. The high antioxidant activity of compounds **1** and **2** is certainly due to the free phenol groups present in each structure. The higher activity of compound **1** compared to that of compound **2** is easily interpreted through the presence of an additional free hydroxyl group at the 7 position which could easily transfer its hydrogen atom to DPPH. These two flavonoids remain less active than their analogue quercetin (5.00 ± 0.12 μg/mL) used as positive control. The significant activity of these phenolic derivatives is in good agreement with literature data [20]. However, compound **1** was found to be more active against DPPH than its aglycon (isorhamnetin) showing an IC_50_ value of 24.5 μg/mL [21]. Similarly, compound **2** exhibited a better antioxidant activity towards DPPH than its aglycon (rhamnetin, IC_50_ = 140 μg/mL) [22]. These findings showed clearly the contribution of the sugar moiety in compounds **1** and **2** either by improving the solubility of these compounds in ethanol or by participating in the stability of the new radical that forms after the transfer of the hydrogen atom.

#### 2.3.2. ABTS Radical Scavenging Activity

ABTS^+^ is a well-known nitrogen-centered synthetic radical and is also used to evaluate antioxidant potential. The ABTS^+^ radical is generated by oxidation of ABTS with potassium persulphate and, when samples are added to the ABTS^+^ radical, it is converted into a non-radical form. The results from the radical scavenger assays using the ABTS^+^ radical for the prepared extracts, fractions and the isolates **1** and **2**, expressed as IC_50_ (μg/mL), are shown in Table 4. These results showed that the different samples tested reacted with ABTS^+^ radical as they have practically done with DPPH. All the samples exhibited interesting radical-scavenging activity towards the ABTS^+^ radical with IC_50_ values ranging from 17.03 ± 0.02 μg/mL (compound **1**) to 44.00 ± 1.72 μg/mL (EtOAc fraction of *D. virgata* flowers) compared to BHT (50.00 ± 0.20 μg/mL).

Indeed, the n-BuOH fraction from *D. virgata* flowers was the most potent according to ABTS radical-scavenging activity with IC_50_ value of 25.54 ± 1.32 μg/mL whereas the crude ethanolic extract of the non-flowering aerial parts of *D. erucoides* was found to be slightly more active (27.04 ± 1.53 μg/mL) than its fractions (EtOAc and n-BuOH). This result can be interpreted by a possible synergism effect due to the concentration of some phenolic components. On the other hand, the results showed that compounds **1** **(**17.03 ± 0.02 μg/mL) and **2** (19.04 ± 0.02 μg/mL) are more than twice as active as BHT. According to ABTS radical-scavenging activity, compounds **1** and **2** remain also less active than quercetin (6.91 ± 0.42 μg/mL).

**Table 4:** Antioxidant activity (DPPH, ABTS^+^) of extracts and compounds **1** and **2** from *Diplotaxis* *virgata* and *D*. *erucoides* expressed as IC_50_ (μg/mL).

Samples	DPPH Radical ^a^	ABTS^+^ Radical ^a^
	IC_50_^b^	IC_50_
Flowers(D. *virgata*)		
EtOH extract	26.03 ± 1.42	31.80 ± 1.12
EtOAc fraction	40.05 ± 1.78	44.00 ± 1.72
n-BuOH fraction	20.01 ± 0.32	25.54 ± 1.32
Compound **1**	16.01 ± 0.13	17.03 ± 0.02
Aerial parts(D. *erucoides*)		
EtOH extract	27.02 ± 1.53	27.04 ± 1.53
EtOAc fraction	26.03 ± 1.61	32.06 ± 1.63
n-BuOH fraction	24.01 ± 1.02	28.05 ± 1.43
Compound **2**	18.00 ± 0.01	19.04 ± 0.02
BHT ^c^	18.00 ± 0.23	50.00 ± 0.20
Quercetin ^d^	5.00 ± 0.12	6.91 ± 0.42

^a^ Values were expressed as mean ± SE (n = 3). ^b^ IC_50_ (μg/mL): the concentration at which 50% is inhibited. ^c^ BHT: butylated hydroxytoluene (reference). ^d^ reference.

### 2.4. Antibacterial Activity

The antibacterial activity of the n-BuOH fractions of *Diplotaxis virgata* and *D.* *erucoides* as well as of the isolated compounds **1** and **2** was assessed *in vitro* against a panel of Gram + (*Listeria monocytogenes* and *Staphylococcus aureus*) and Gram–(*Aeromona hydrophila*, *Pseudomonas aeruginosa*, *Salmonella enteritidis*, *Escherichia coli* and *Klebsiella pneumoniae*) bacteria by using the disc diffusion method. The results are given in Table 5. According to these results, all the tested samples exhibited an interesting antibacterial activity against most bacteria tested. The antimicrobial activity of our n-BuOH extracts is comparable to that of extracts from *Diplotaxis harra* and *D. Simplex* [19]. The n-BuOH extract of *D. virgata* flowers was found to be more effective (IZ = 15.00 ± 0.02–22.03 ± 0.02 mm) against all the used bacteria than the isolated compound **1** (IZ = 12.04 ± 0.05–16.55 ± 0.05 mm). This finding may be explained by a possible synergism effect due to the concentration of several antibacterial compounds present in this extract. *E. coli* appeared the most sensitive towards the n-BuOH extract of *D. virgata* flowers (IZ = 22.03 ± 0.02 mm) followed by *A. hydrophila* (IZ = 21.00 ± 0.05 mm) (Table 5).

The used bacteria were found to be more sensitive towards the n-BuOH extract of *D. erucoides* aerial parts (IZ = 16.00 ± 0.01–21.07 ± 0.05 mm) than compound **2** (IZ = 13.00 ± 0.02–17.60 ± 0.04 mm) except *E. coli* which was slightly more sensitive against compound **2** (IZ = 17.60 ± 0.04 mm). Additionally, compound **2** was slightly more efficient than compound **1** against all bacteria tested. This finding could be explained essentially by the additional sugar moiety in compound **2** at position C-3ʹ. Tsuchiya and colleagues sought to establish a structure–activity relationship for flavonoids by the isolation of a number of differently substituted compounds and testing their antibacterial activity towards a panel of bacterial strains. Their study demonstrated that 5,7-dihydroxylation of the A ring in the flavonoid skeleton was important for antibacterial activity [23]. This results reinforce the antibacterial effect of compounds **1** and **2**, dihydroxylated, hydroxylated and methoxylated in those positions, respectively.

**Table 5:** Antibacterial activity of n-butanol fractions and compounds **1** and **2** from *Diplotaxis virgata* and *D. erucoides*.

Bacterial Strains	Inhibition Diameter ^a^ (IZ in mm), Concentration = 250 µg/mL				
	n-BuOH (*D. virgata*)	Compound 1	n-BuOH (*D. erucoides*)	Compound 2	Ampicillin ^b^
**Gram + Bacteria**					
*Listeria monocytogenes* ATCC 11120	20.00 ± 0.01	13.60 ± 0.04	16.02 ± 0.03	14.50 ± 0.01	16.00 ± 0.01
*Staphylococcus aureus* ATCC 25923	19.04 ± 0.05	12.04 ± 0.05	20.04 ± 0.01	13.00 ± 0.02	18.00 ± 0.03
**Gram − Bacteria**					
*Aeromona hydrophila* ATCC 1943	21.00 ± 0.05	16.00 ± 0.01	21.07 ± 0.05	17.50 ± 0.05	17.00 ± 0.02
*Pseudomonas aeruginosa* ATCC 9027	15.00 ± 0.02	13.00 ± 0.03	20.00 ± 0.02	17.00 ± 0.05	19.00 ± 0.01
*Salmonella enteritidis* ATCC 14028	15.03 ± 0.01	12.50 ± 0.05	18.00 ± 0.02	14.00 ± 0.03	19.00 ± 0.04
*Escherichia coli* ATCC 25922	22.03 ± 0.02	16.55 ± 0.05	16.00 ± 0.01	17.60 ± 0.04	20.00 ± 0.03
*Klebsiella pneumonia* ATCC 13833	16.00 ± 0.03	14.00 ± 0.01	17.06 ± 0.02	15.00 ± 0.02	21.00 ± 0.02

^a^ Values were expressed as mean ± SE (n = 3). Internal diameter = 6 mm, inhibitions diameter = external diameter−internal diameter. ^b^ antibiotic (reference).

## 3. Experimental Section

### 3.1. General Experimental Procedures

UV spectra and Antioxidant activity was measured on a UV**-**Vis Jenway 6505 spectrophotometer. NMR experiments were performed on a BRUKER AVANCE *III* HD 400 spectrometer operating at 400.13 MHz equipped with a QNP ^1^H/ ^13^C/ ^19^F/ ^31^P probe. Spectra were recorded at 30 °C in a mixture of deuterated solvents (0.5 mL CD_3_OD, 0.3 mL D_2_O). Acquisition sequences were 1D-NMR ^1^H-, ^13^C-, DEPT 135 and 2D-NMR experiments COSY, HSQC, HMBC, and NOESY. The chemical shifts (μ) reported in ppm were referred to residual non deuterated CH_3_OH. Coupling constants were measured in Hz and signals are using the following abbreviations: s, singlet; d, doublet; dd, doublet of doublets; m, multiplet. HPLC analysis (Agilent 1100), was carried out on a YMC**-**Pack Pro C18 column (3.0 × 150 mm, 3.5 µm) under the following conditions: injected volume 1 µL, flow rate 0.3 mL/min, column temperature 40 °C, wavelength detection at 210 nm (Bw: 10, Slit 2 nm); 254 nm (Bw: 2, Slit 2 nm); and 340 nm (Bw: 8, Slit 2 nm). A gradient elution was carried out using the following solvent system: mobile phase A, water formic acid (99.9: 0.1 (v/v)); mobile phase B, methanol. The gradient program (flow 0.3 mL/min) was from 10%–100% methanol as followed: 10% (0–1 min); 100% (1–10 min) as post run time 4 min. MS analysis was carried out using a quadrupole time of flight (QTOF) mass detector (microToF-Q*II*-Bruker Daltonics), equipped with an electrospray interface operating in positive and negative ion mode, using the following operation parameters: capillary voltage 3500 V, nebulizer pressure 0.6 bar, drying gas 4 L/min, gas temperature 180 °C. MS accurate mass spectra were recorded across the range 50–1000 *m*/*z*.

### 3.2. Plant Material

*Diplotaxis virgata* (L.) D.C. was collected from the region of Sousse (Tunisia) and *Diplotaxis* *erucoides* (L.) D.C. was collected from the region of Zaghouan (Tunisia) in March 2012, and identified by Prof. Nadia Ben BRAHIM (National Agricultural Research Institute of Tunis). Flowers were separated from aerial parts before extraction. A voucher specimen from each species (DV-12 and DE-12, respectively) has been deposited in the department of Botany of the same Institute.

### 3.3. Extraction and Isolation

The non-flowering aerial parts of *Diplotaxis erucoides* (50 g) and flowers of *Diplotaxis virgata* (50 g) were extracted for three times with 80% ethanol at room temperature for 72 h. The ethanol extracts concentrated under reduced pressure, giving (10 g (20%), 2.5 g (5%), respectively). The residue was successively partitioned with hexane, chloroform, ethyl acetate, n**-**butanol and water. The extracted solutions were evaporated under reduced pressure to yield 320 mg (3.2%), 300 mg (12%), of a hexane**-**soluble extract, 140 mg (1.4%), 160 mg (6.4%), of CHCl3-soluble extract, 379 mg (3.79%), 180 mg (7.2%) of EtOAc**-**soluble extract, 1.179 g (11.79%), 0.950 g (38%) of butanol**-**soluble extract, respectively and the remaining aqueous solution were lyophilized to yield 7.98 g (79.8%), 0.91 g (36.4%) of water**-**soluble extract, respectively.

The n**-**BuOH extract of flowers of *D*. *virgata* (250 mg) was fractionated by HPLC**-**UV on a C18 SPE cartridge using as solvent water formic acid 99.9:0.1 (v/v) (A; 100%–0%) and MeOH (B; 0%–100%). A total of 10 fractions were obtained. Fraction eluted by (50% A; 50% B) constitutes compound **1** (25 mg). The fractionation of the n-BuOH extract of the non-flowering aerial parts of *D. erucoides* (250 mg) by HPLC**-**UV on a C18 SPE cartridge applying the same mobile phase, gives 10 fractions. The purified fraction using (50% A; 50% B) corresponds to compound **2** (23 mg).

### 3.4. Fatty Acid Determination (GC-MS)

The process of converting free or esterified FAs into methyl esters is referred to as transesterification, with a methanolic solution of potassium according to the AOAC official method 969.33 [24]. Individual FAMEs were separated and quantified by gas chromatography. GC analysis was carried out on Agilent 6890N Network GC system combined with agilent 5973 Network Mass Selective Detector (GC**-**MS). The capillary column used was an agilent 19091N**-**205 (HP Innowax capillary; 50.0 m × 200 µm; 0.4 µm). Helium was used as carrier gas at a flow rate of 0.8 mL/min with 1 µL injection volume. Samples were analyzed with the column held initially 60 °C for 2 min after injection then 5 °C/min to 250 °C for 20 min. The injection was performed in splitless mode. Dectector and injector temperature were respectively 280 and 250 °C. Run time was 80 min. MS scan range was (*m*/*z*) 35–450 atomic mass units (a.m.u) under electron impact (EI) ionization (70 eV).

### 3.5. Antioxidant Activity

#### 3.5.1. DPPH Assay

This test allows to measure the extracts capacity to scavenge the stable radical 2,2-diphenyl-1-picryl hydrazil (DPPH) formed in solution by donation of a hydrogen atom or an electron [25]. If the extracts have the capacity to scavenge the DPPH free radical, the initial blue/purple solution will change to yellow due to diphenylpicrylhydrazine formation. This reaction is used as a measure of the extracts ability to scavenge any free radical. An amount of 0.5 mL of each sample concentration was mixed using the same volume of DPPH ethanolic solution. After an incubation of 30 min in darkness and at a temperature of 25 °C, absorption was read at 517 nm wavelength. A mixture of 0.5 mL of DPPH solution and 0.5 mL of ethanol was taken as a blank [26]. The synthetic BHT and quercetin were used as positive control. The decrease in absorption induced by the tested samples was compared to that of the positive control BHT. The calculated IC_50_ values denote the concentration required to scavenge 50% of DPPH radicals. Results were expressed in inhibition percentage at different sample concentrations (mg/mL). The inhibition of free radical DPPH in percentage (I%) was calculated as follows:

(1) I% = ((A _blank_ − A _sample_)/A _blank_) × 100

where A _blank_ is the absorbance of the control reaction (containing all reagent except the test compound) and A _sample_ is the absorbance of the test compound. Extract concentration providing 50% inhibition percentage against extract concentration. Tests were carried out in triplicate.

#### 3.5.2. ABTS Test

The antiradical activity was done using the ABTS^+^ free radical decolorization assay developed by the method of [27], with some modifications. Briefly, the ABTS preformed radical monocation was generated by reacting ABTS solution (7 mM) with 2.45 mM K_2_S_2_O_8_. The mixture was allowed to stand for 15 h in the dark at room temperature. The solution was diluted with ethanol to obtain the absorption of 0.7 ± 0.2 units at 734 nm. Samples were separately dissolved in ethanol to yield the following concentrations: (0.0312, 0.0625, 0.125, 0.25, 0.5, and 1 mg/mL). In order to measure the extracts antioxidant activity, 10 µL of each were added to 990 µL of diluted ABTS^+^ at various concentrations. The absorption was read after 20 min. The synthetic BHT and quercetin were used as positive control. The antioxidant capacity of the tested samples was expressed as the inhibition percentage (%). The ABTS^+^ scavenging percentage was calculated according to the following formula:

(2) ABTS scavenging activity (%) = [(A_blank_ − A_sample_)/A_blank_)] × 100

where A_blank_ is the absorbance of the control reaction (containing all reagents except the test compound), and A_sample_ is the absorbance of the test compound.

### 3.6. Antimicrobial Activity by Wells Method

The *in vitro* antibacterial activity of the tested samples was carried out by wells agar diffusion method [28], against 2 Gram-positive bacteria (*Staphylococcus aureus* ATCC 25923 and *Listeria monocytogenes* ATCC 11120) and 5 Gram-negative bacteria (*Pseudomonas aeruginosa* ATCC 9027, *Salmonella enteritidis* ATCC 14028, *Escherichia coli* ATCC 25922, *Aeromonas hydrophila* ATCC 1943 and *Klebsiella pneumoniae* ATCC 13833). The nutrient agar was used as culture medium. One hundred microliters of each tested strain (about 10^7^ CFU/mL) was added to 20 mL of molten medium and let to solidify in sterile petriplates. After solidification, wells were perforated using a sterile cork borer (6.0 mm diameter) and 50 µL of n-BuOH extract or pure compound (250 µg/mL) were added. The plates were incubated at 37 °C and examined after 24 h for clear zones showing the bacteria inhibition. The negative control plates had no product added to the filter paper whereas in the positive control plates, discs were impregnated with the same volume of ampicillin solution (5 mg/mL). The diameters of the inhibition zones were measured. The experiments were repeated in triplicates and the results were expressed as average values.

## 4. Conclusions

In summary, this work described the chemical investigation of two Tunisian *Diplotaxis* species, *D. virgata* and *D. erucoides,* which led to the isolation of two new flavonoids, isorhamnetin**-**3**-***O***-**α**-**l**-**glucopyranoside (**1**) and rhamnetin**-**3,3ʹ**-**di**-***O***-**β**-**d**-**glucopyranoside (**2**). Their structures were established by UV, HRMS and RMN analysis. The fatty acid composition of the hexane extracts from the two species was determined by gas chromatography (GC-MS) for the first time. The *in vitro* antioxidant activity of the prepared extracts and the isolates was investigated by using DPPH and ABTS^+^. The results demonstrated that the n-BuOH extracts and the isolated compounds exhibit potent free radical scavenging activities. These antioxidant activities were found to be in perfect harmony with the structure of the identified phenolic compounds. The n-BuOH extracts from the two species and the isolated compounds **1** and **2** were also tested for their inhibitory effects against a panel of bacterial agents and it was found that compounds **1** and **2** exhibited interesting antibacterial activity toward most bacteria tested (IZ = 12.04 ± 0.05–17.60 ± 0.04 mm). Finally, this research work reinforced the potential of these species as a possible source of novel bioactive compounds and allowed the discovery of new bioactive flavonoids.

## Supplementary Materials

Supplementary materials, including 1D (^1^H-, ^13^C-, DEPT 135) and 2D (COSY, HSQC, HMBC, NOESY) NMR and high resolution mass spectrometry spectra for compounds **1** and **2**, can be accessed at: http://www.mdpi.com/1420-3049/20/10/18128/s1.
