# Supplementary material for: Phytochemical and Biological Investigation of Two Diplotaxis Species Growing in Tunisia: D. virgata & D. erucoides
Source: Molecules. 2015 Oct 5;20(10):18128–43. doi: 10.3390/molecules201018128 (PMC6332249; doi:10.3390/molecules201018128)

F7 fraction: Compound 1

Analysis Compound 1 HRMS

Analysis Inf

Analysis Na	O:\Analyses SCA 2014\octobre\Nizar F7.d		Acquisition Date	10/10/2014 12:04:05
Method	MS_Inf_TL_50_1000_pos.m		Operator	BDAL@DE
Sample Nam	Nizar F7	Instrument / Ser#	micrOTOF-Q II	10315
Comment				

Acquisition Param

Source Type	ESI	Ion Polarity	Negative	Set Nebulizer	0.6 Bar
Focus	Not active	Set Capillary	3500 V	Set Dry Heater	180 °C
Scan Begin	50 m/z	Set End Plate Offset	-500 V	Set Dry Gas	4.0 l/min
Scan End	1000 m/z	Set Collision Cell RF	100.0 Vpp	Set Divert Valve	Source

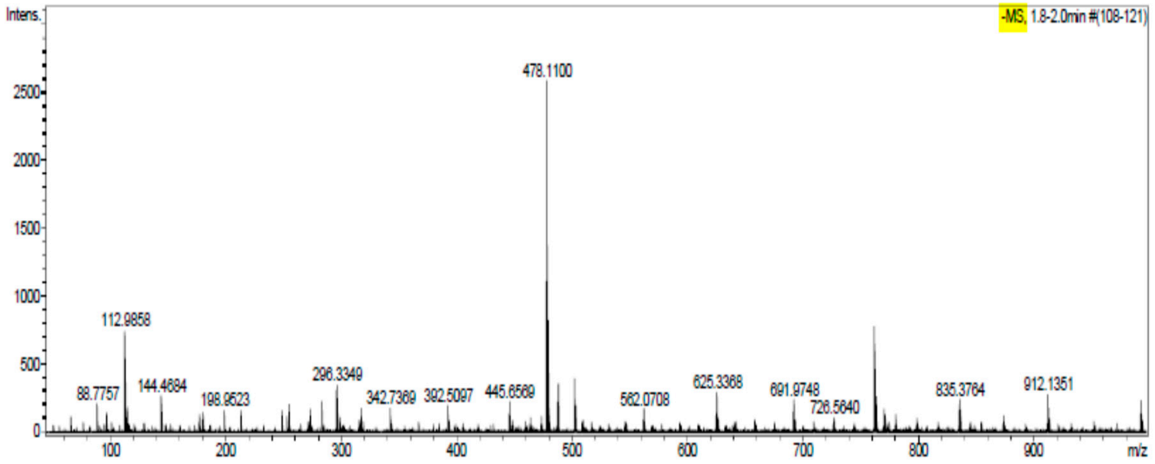

Meas. m/z	#	Formula	Score	m/z	err [mDa]	err [ppm]	mSigma
477.1050	1	C 22 H 21 O 12	100.00	477.1033	-1.7	-3.6	493.6

^{1}H-NMR Spectra

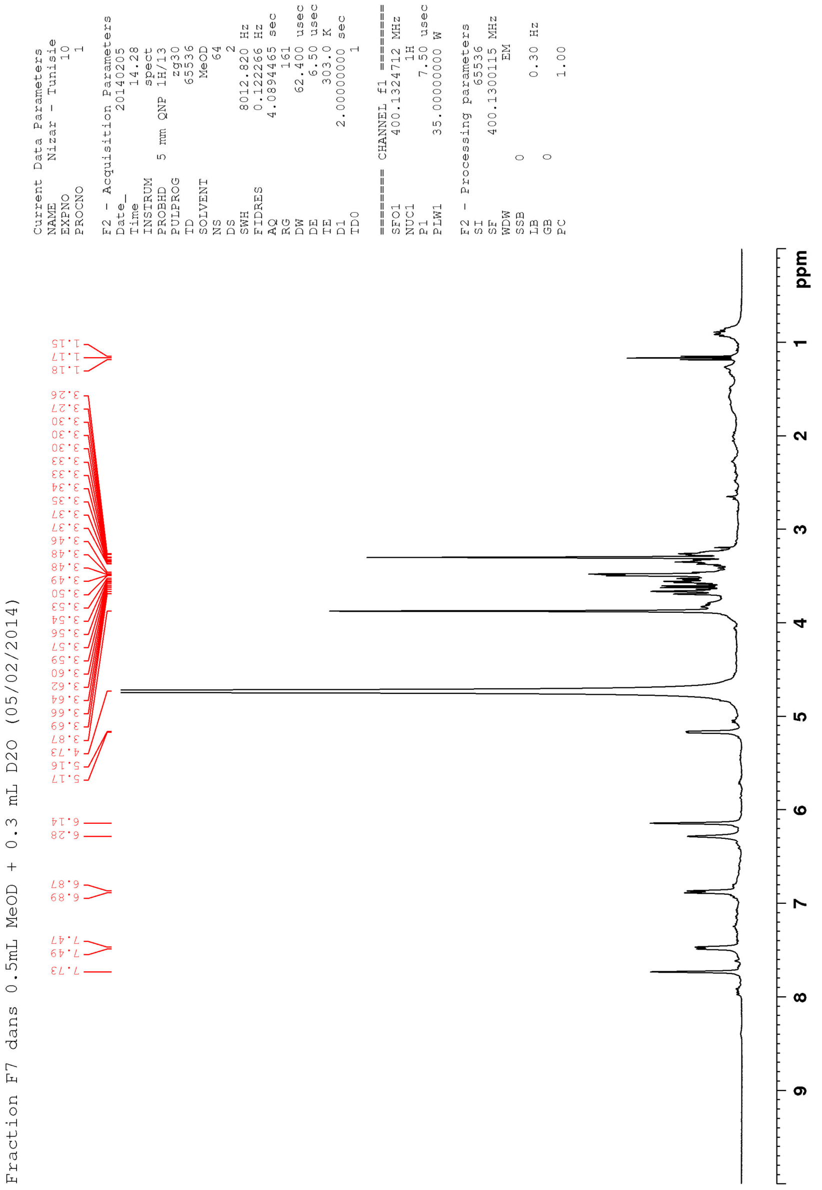

^{13}C-NMR Spectra

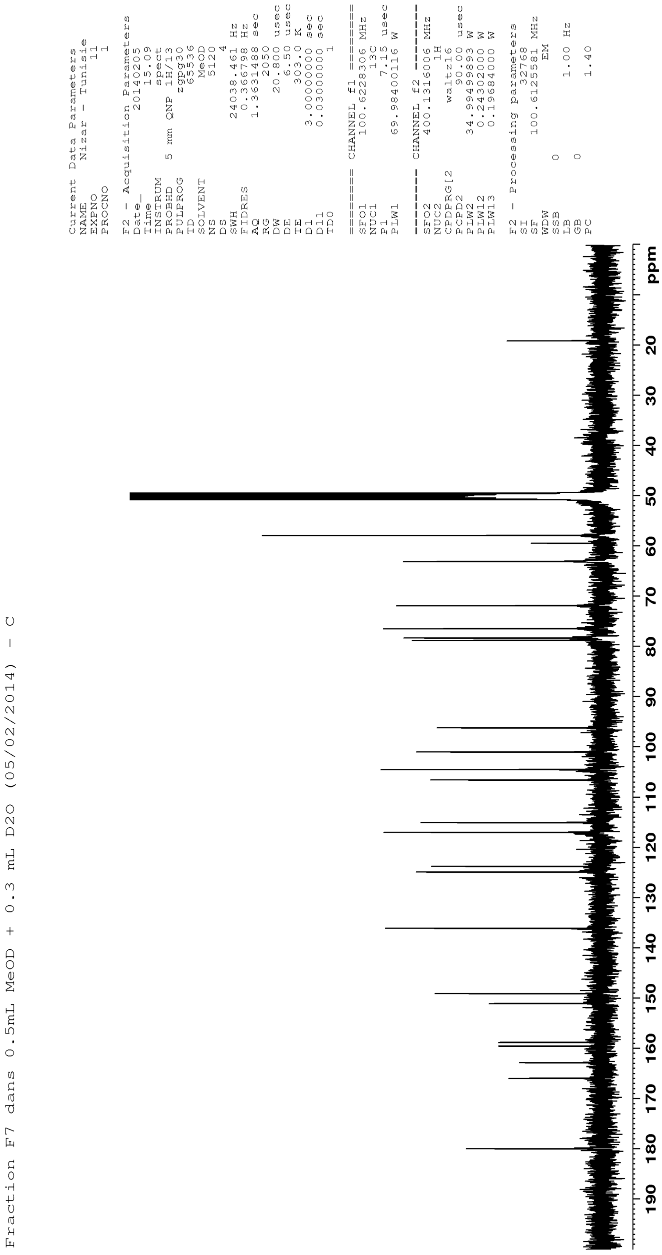

DEPT -90 and DEPT -135 Spectra

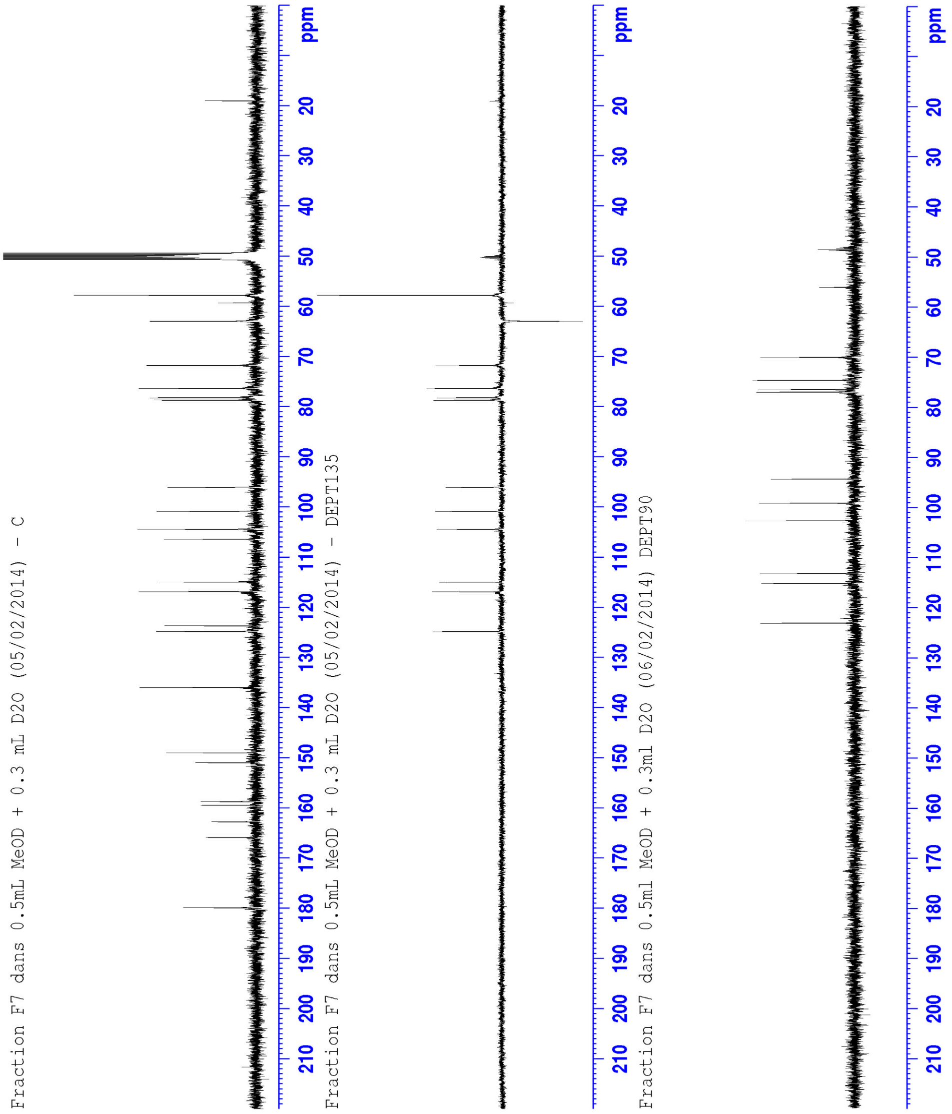

HSQC Spectra

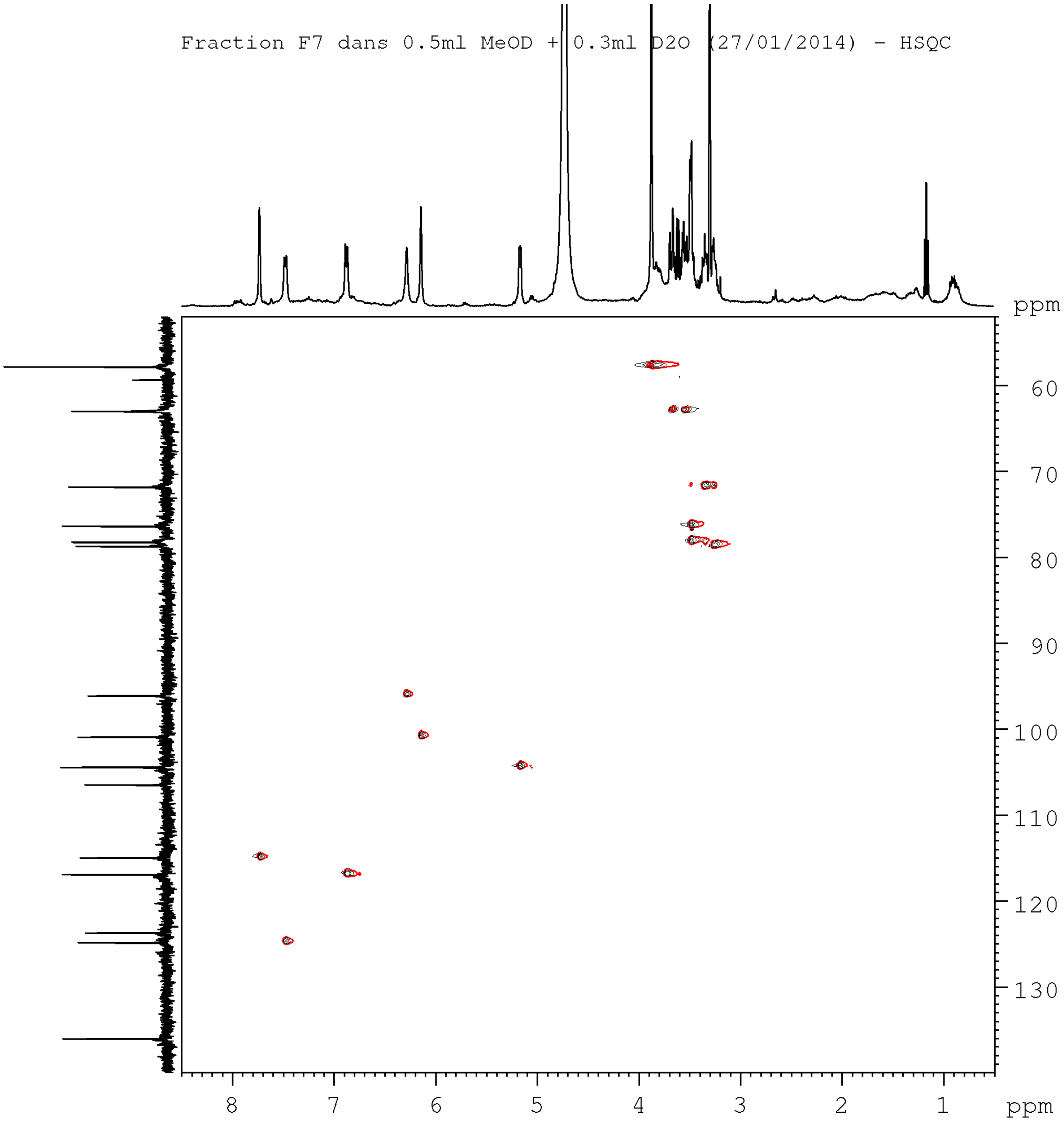

COSY Spectra

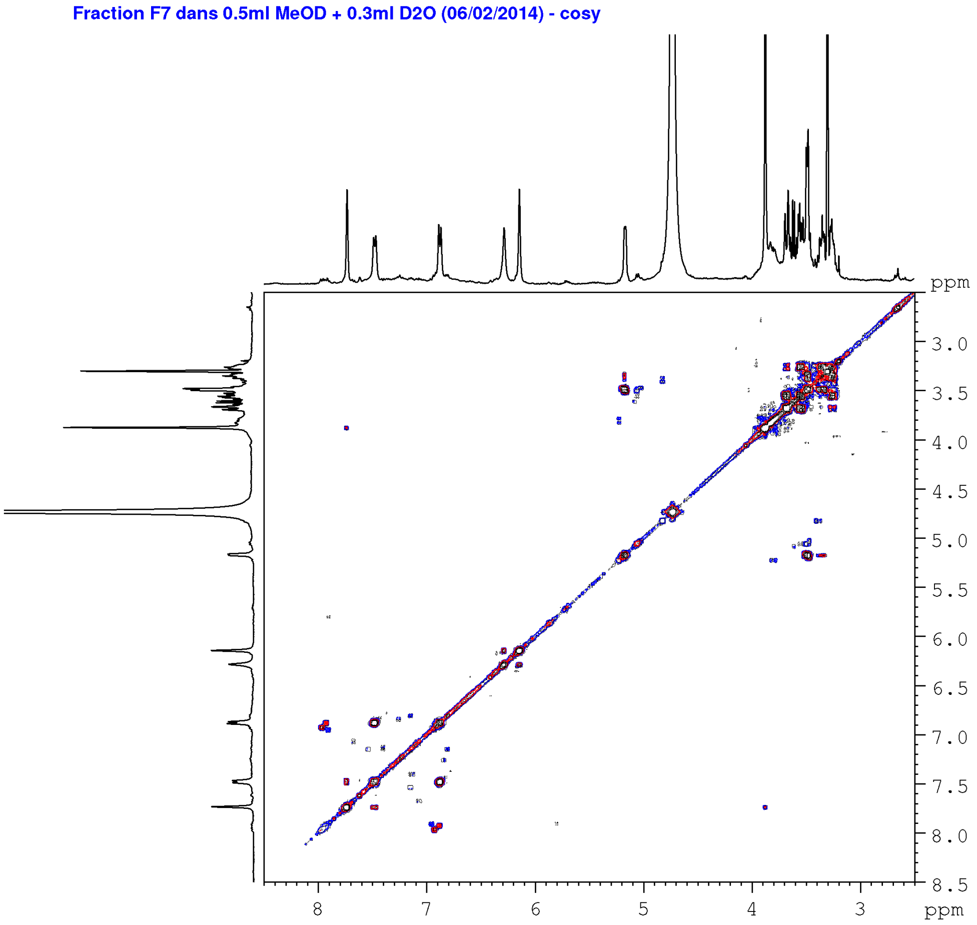

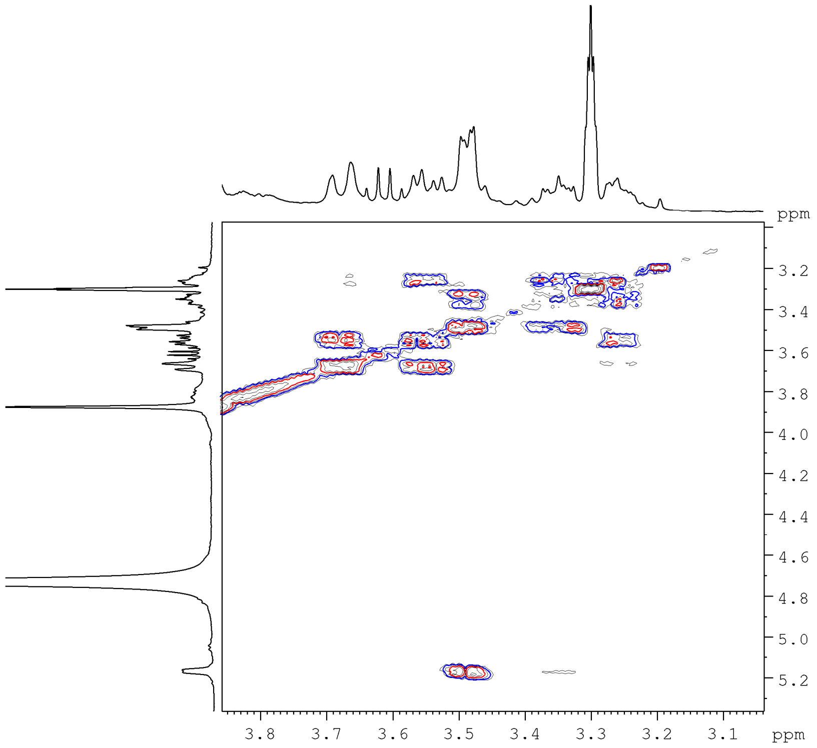

HMBC Spectra

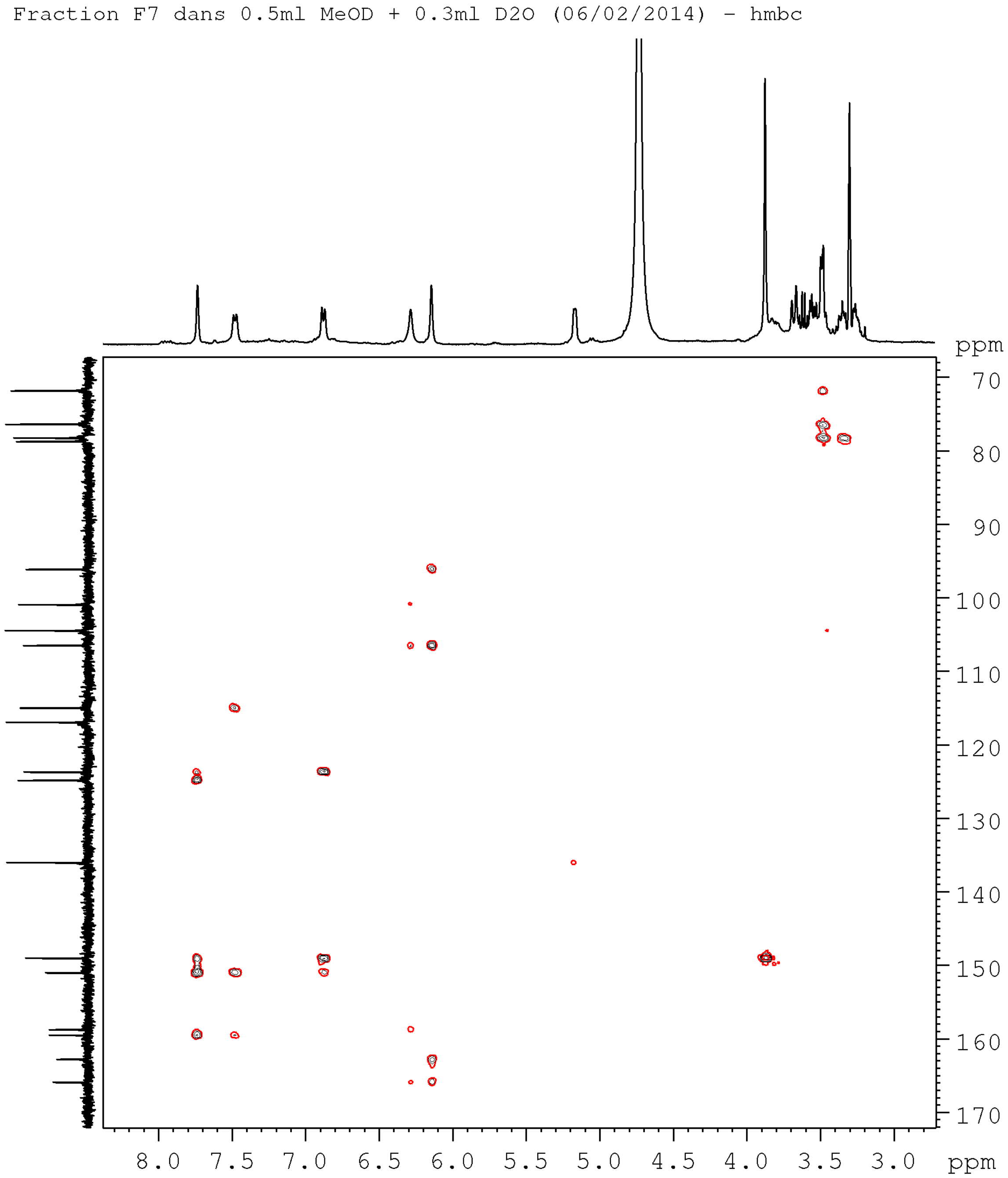

NOESY spectra

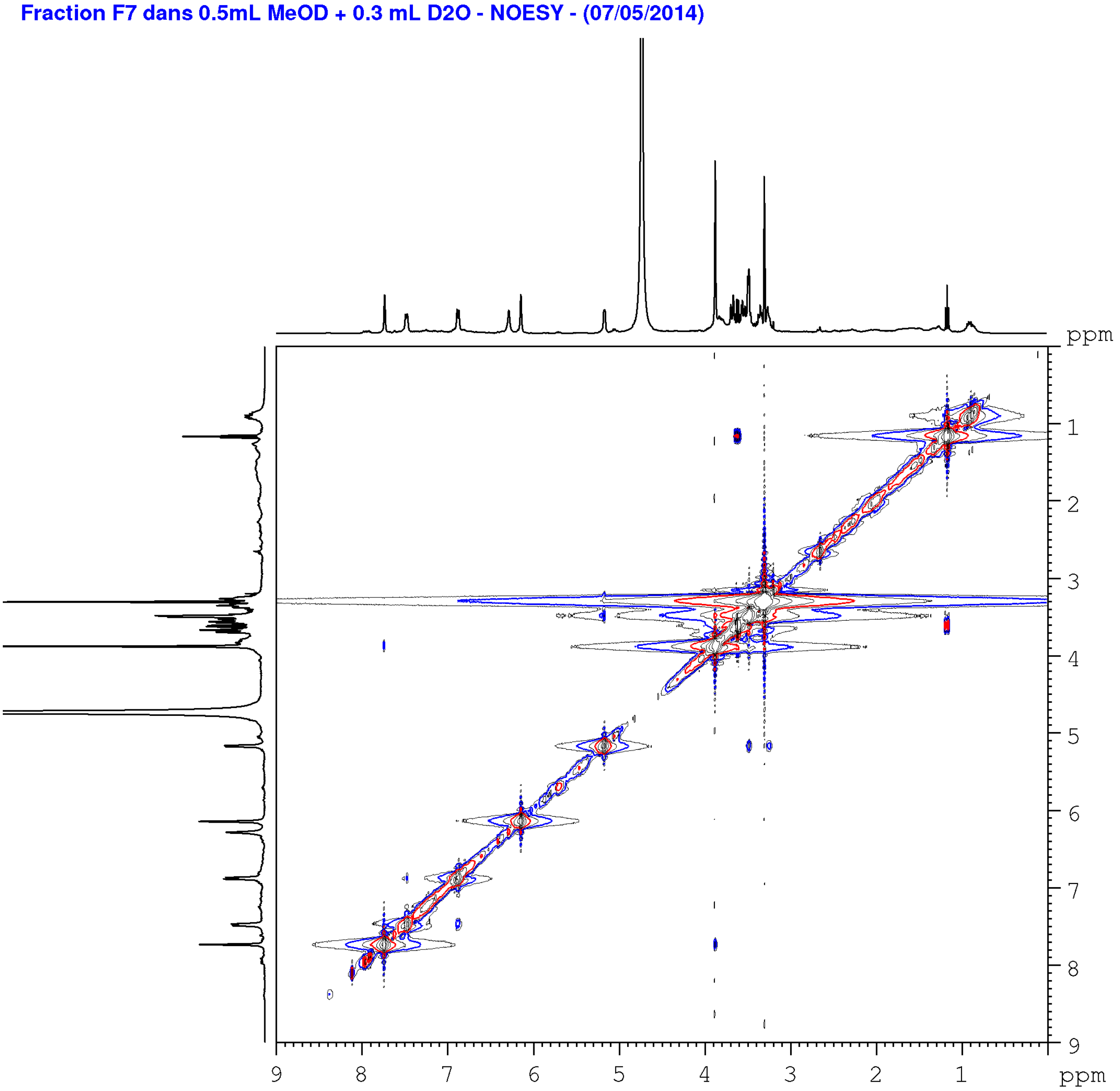

Fraction F7 dans 0.5mL MeOD + 0.3 mL D2O - NOESY - (07/05/2014)

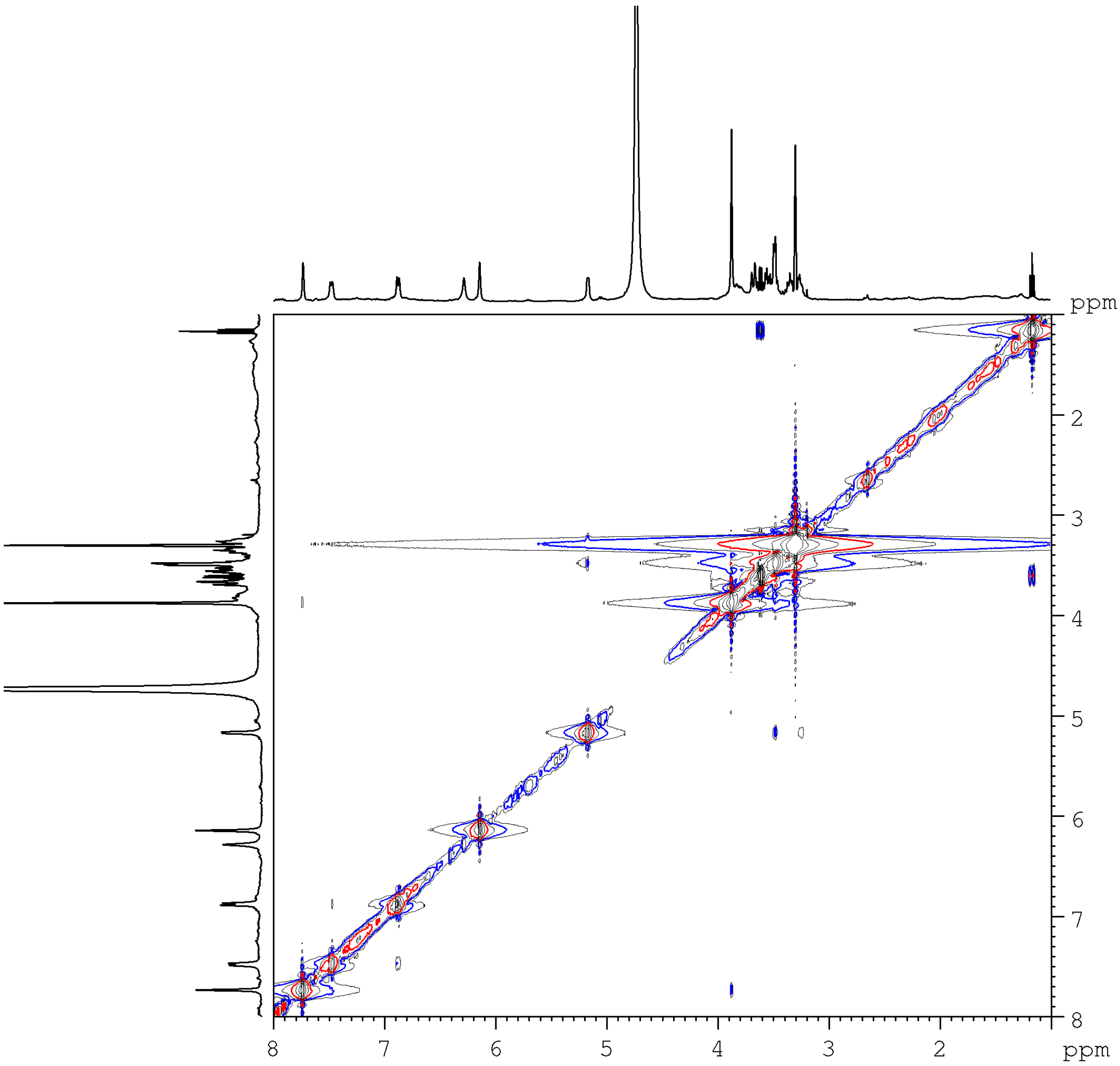

F5 fraction: Compound 2

Analysis Compound 2 HRMS

Analysis Inf

Analysis Na	O:\Analyses SCA 2014\octobre\Nizar F5-2.d		Acquisition Date	10/10/2014 11:58:29
Method	MS_Inf_TL_50_1000_pos.m		Operator	BDAL@DE
Sample Nam	Nizar F5	Instrument / Ser#	micrOTOF-Q II	10315
Comment				

Acquisition Param

Source Type	ESI	Ion Polarity	Negative	Set Nebulizer	0.6 Bar
Focus	Not active	Set Capillary	3500 V	Set Dry Heater	180 °C
Scan Begin	50 m/z	Set End Plate Offset	-500 V	Set Dry Gas	4.0 l/min
Scan End	1000 m/z	Set Collision Cell RF	100.0 Vpp	Set Divert Valve	Source

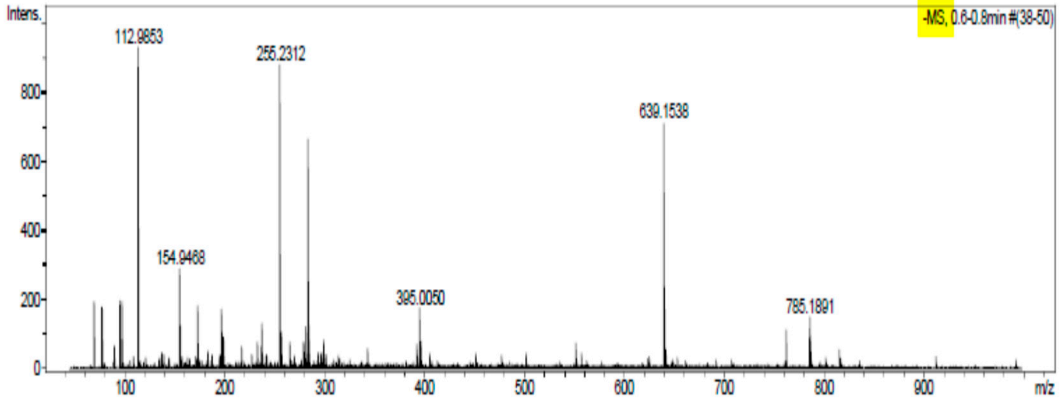

Meas. m/z	#	Formula	Score	m/z	err [mDa]	err [ppm]	mSigma
639.1538	1	C 28 H 31 O 17	100.00	639.1567	2.9	4.5	43.2
	2	C 35 H 27 O 12	66.68	639.1508	-3.0	-4.6	54.6

^{1}H-NMR Spectra

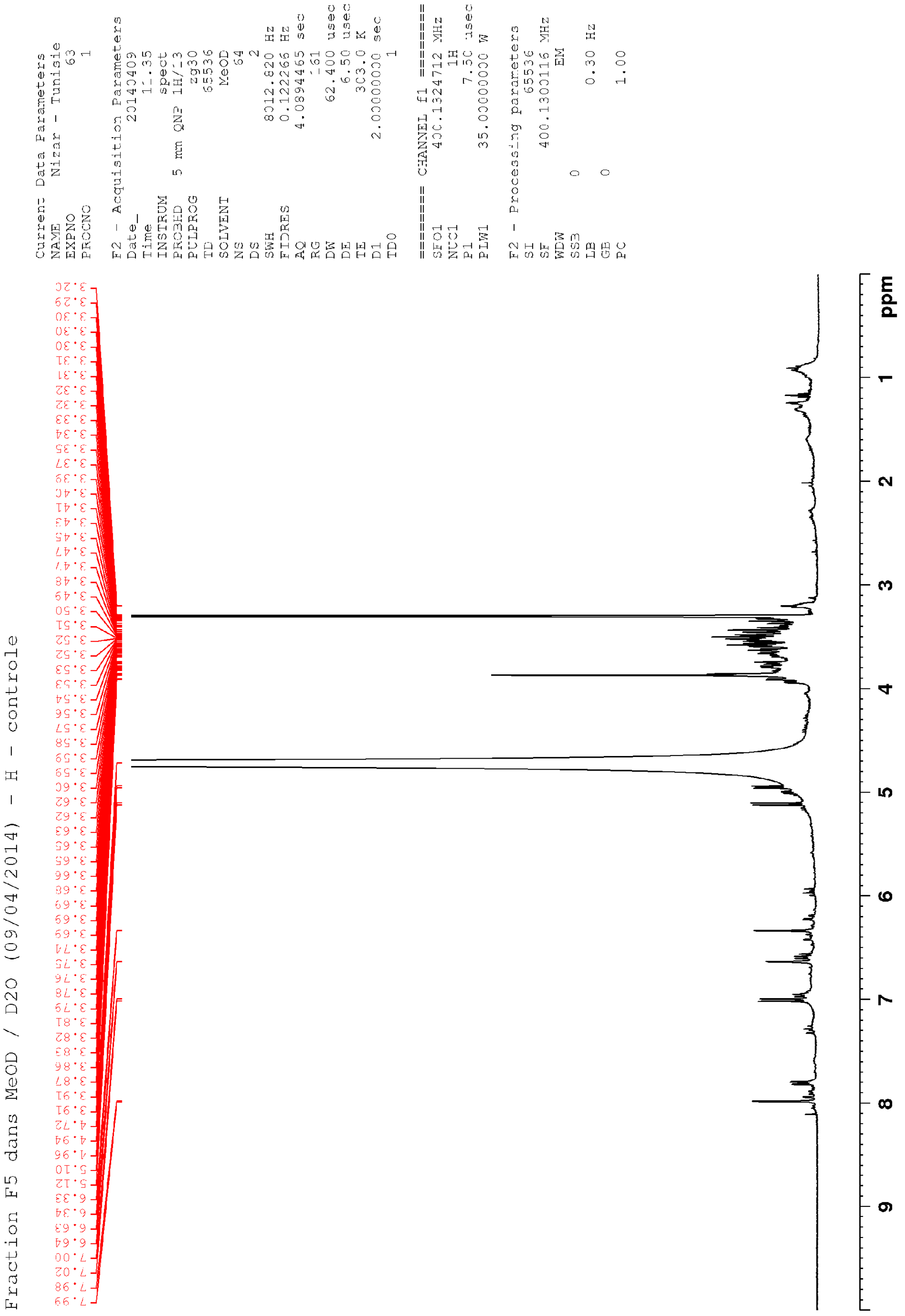

^{13}C-NMR Spectra

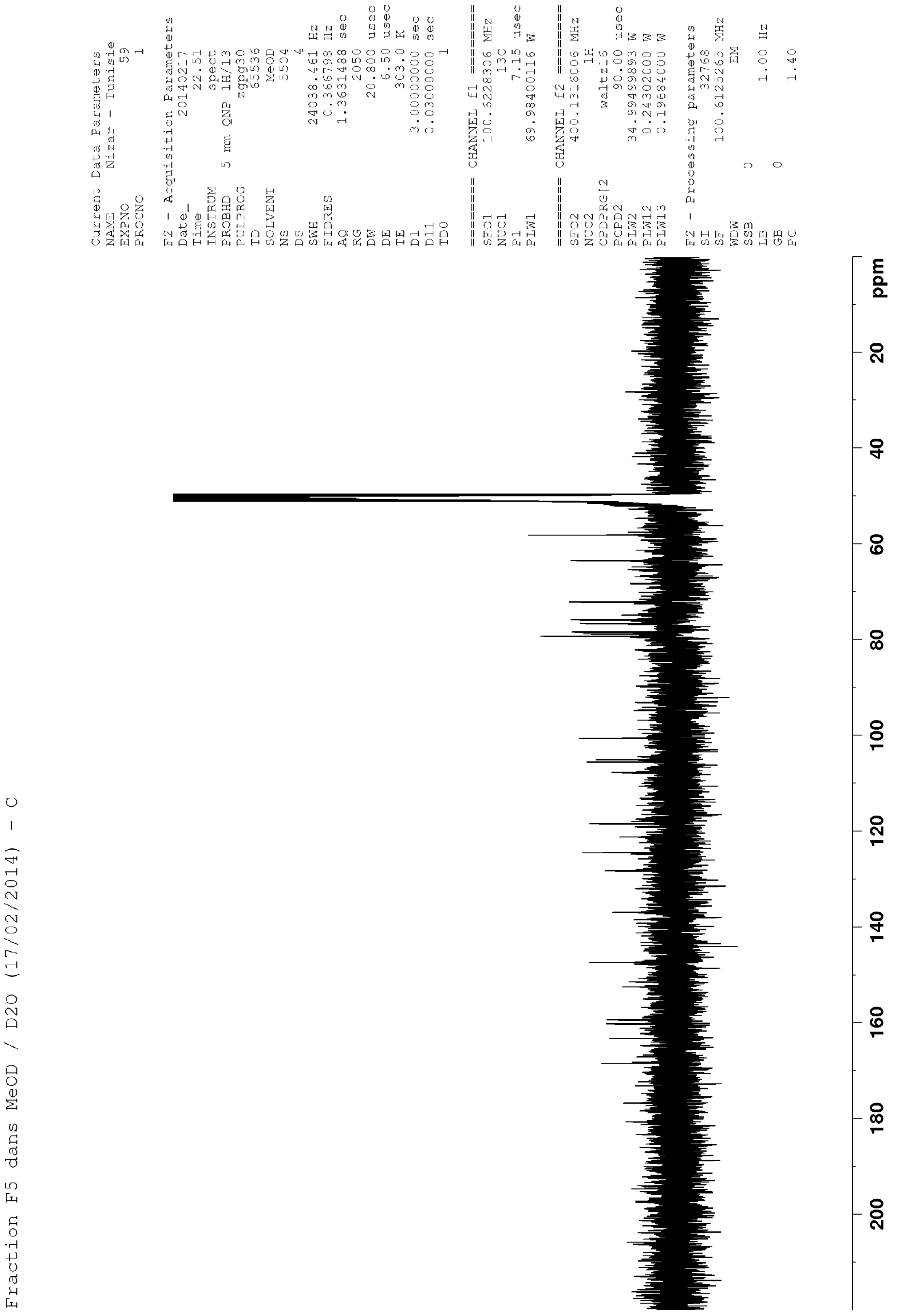

DEPT 135 spectra

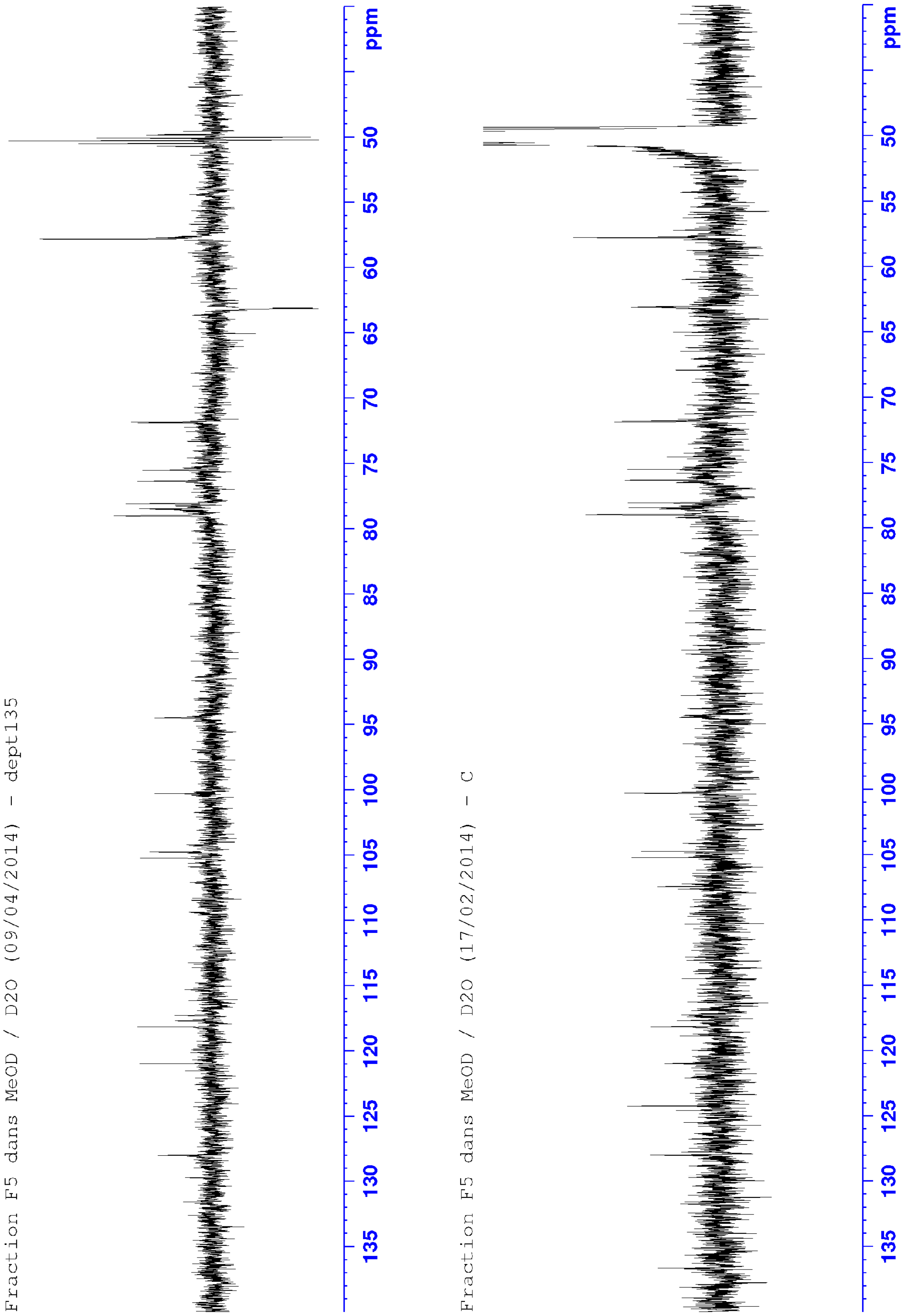

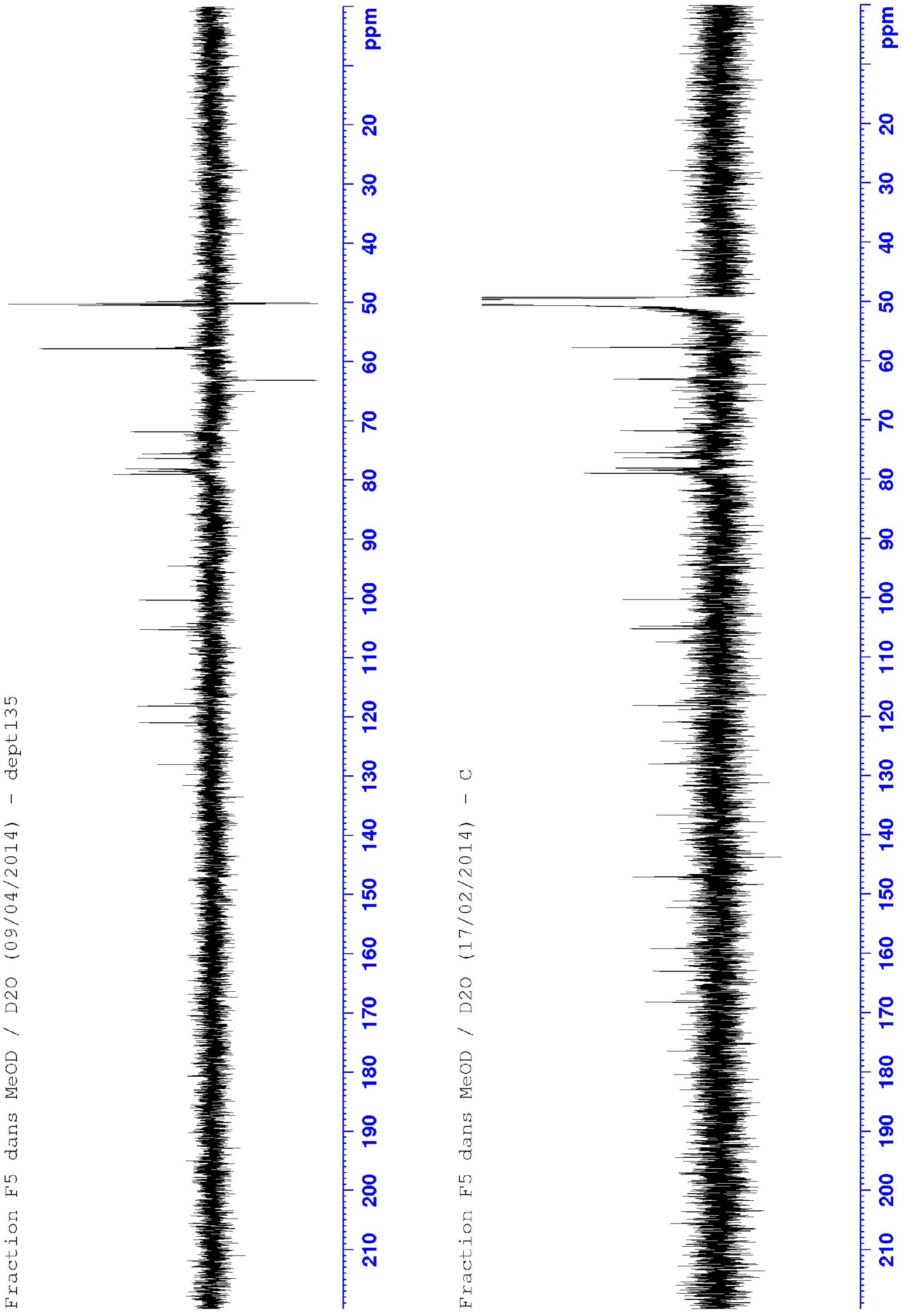
Fraction F5 dans MeOD / D2O (09/04/2014) - dept135
Fraction F5 dans MeOD / D2O (17/02/2014) - C
ppm
ppm

HSQC Spectra

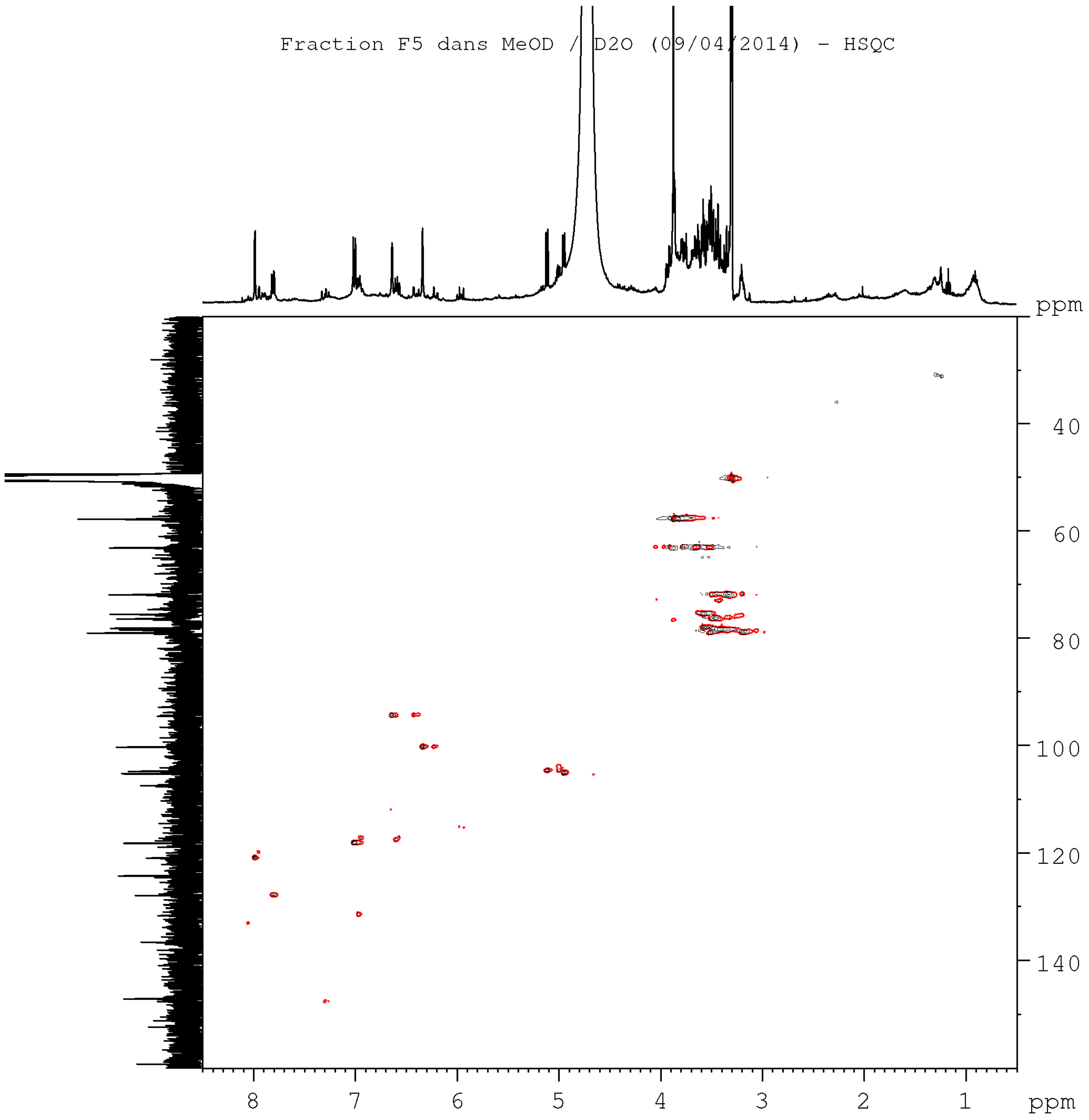

HSQC Spectra

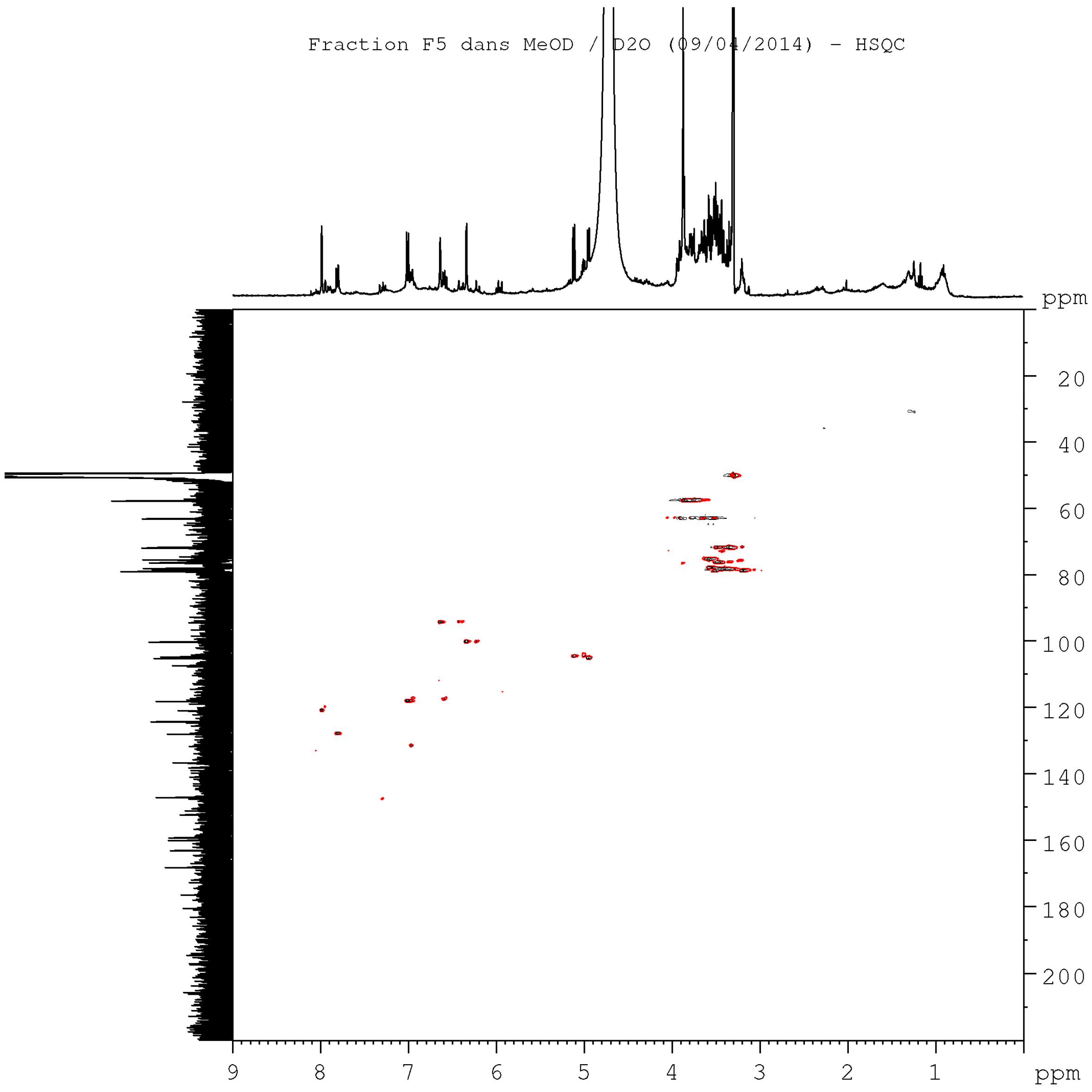

COSY spectra

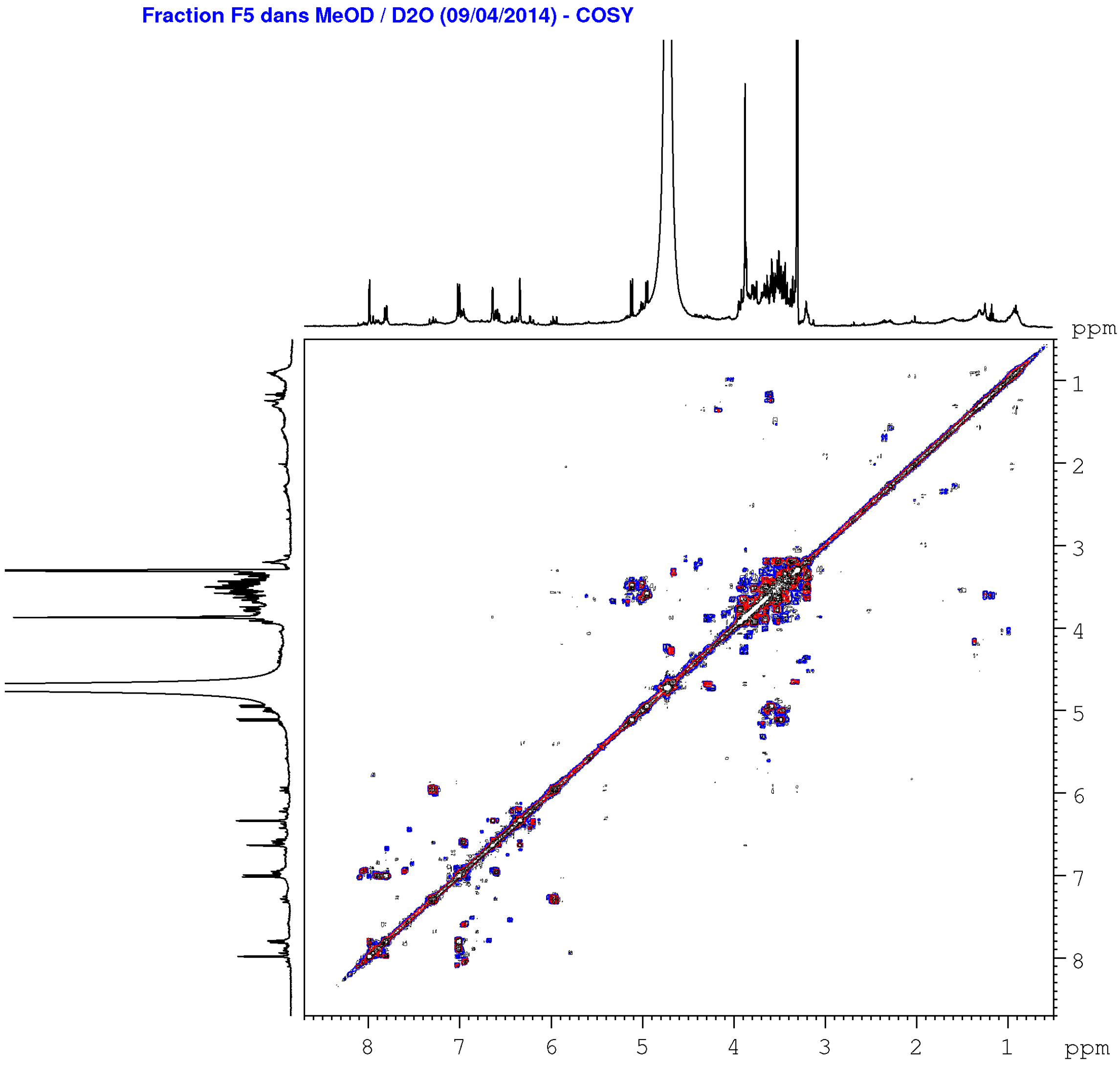

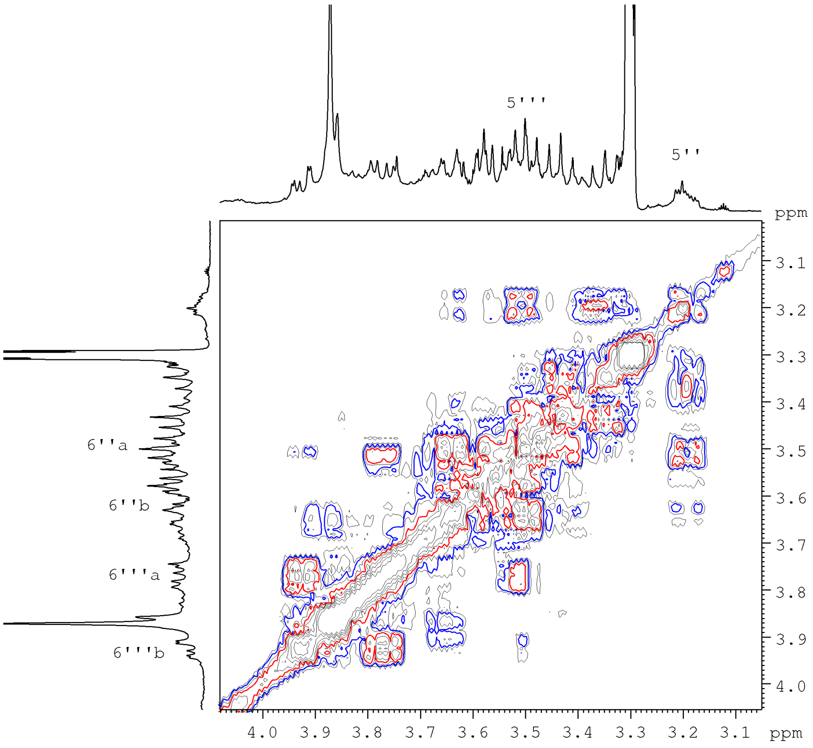
5'''
5''
6''a
6''b
6'''a
6'''b
ppm
3.1
3.2
3.3
3.4
3.5
3.6
3.7
3.8
3.9
4.0
4.0 3.9 3.8 3.7 3.6 3.5 3.4 3.3 3.2 3.1 ppm

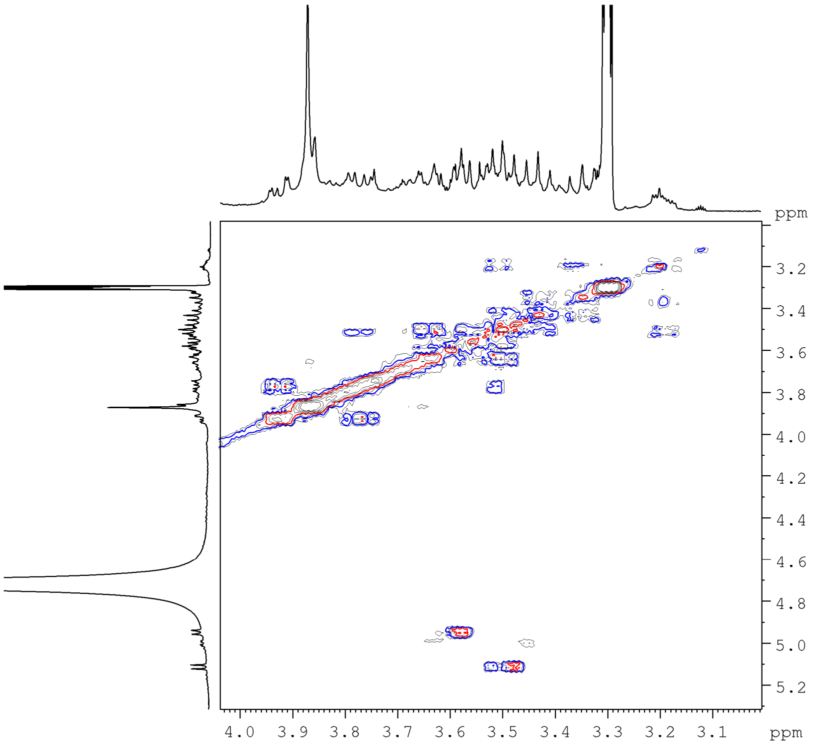
ppm
3.2
3.4
3.6
3.8
4.0
4.2
4.4
4.6
4.8
5.0
5.2
4.0 3.9 3.8 3.7 3.6 3.5 3.4 3.3 3.2 3.1 ppm

HMBC Spectra

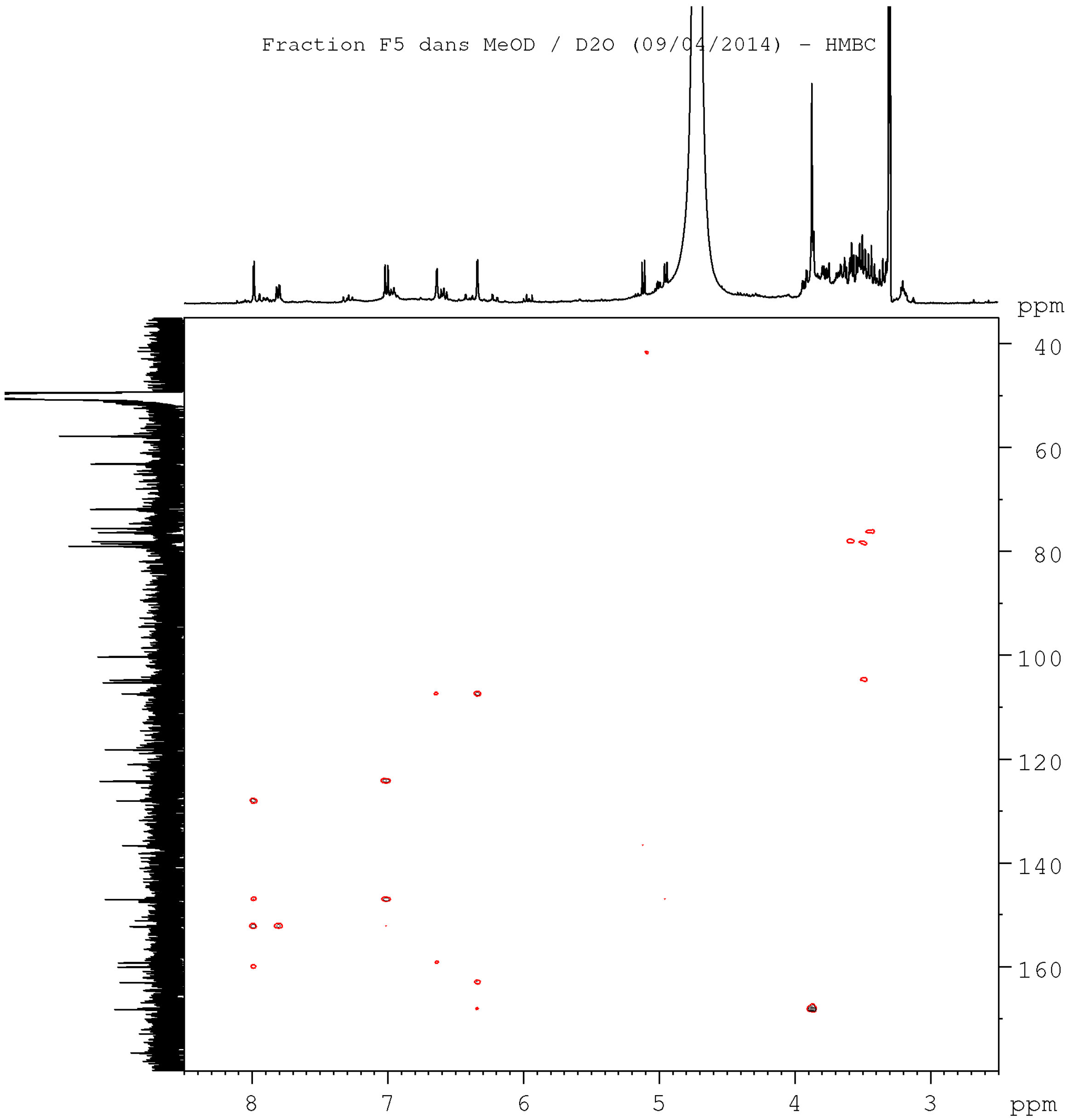

NOESY spectra

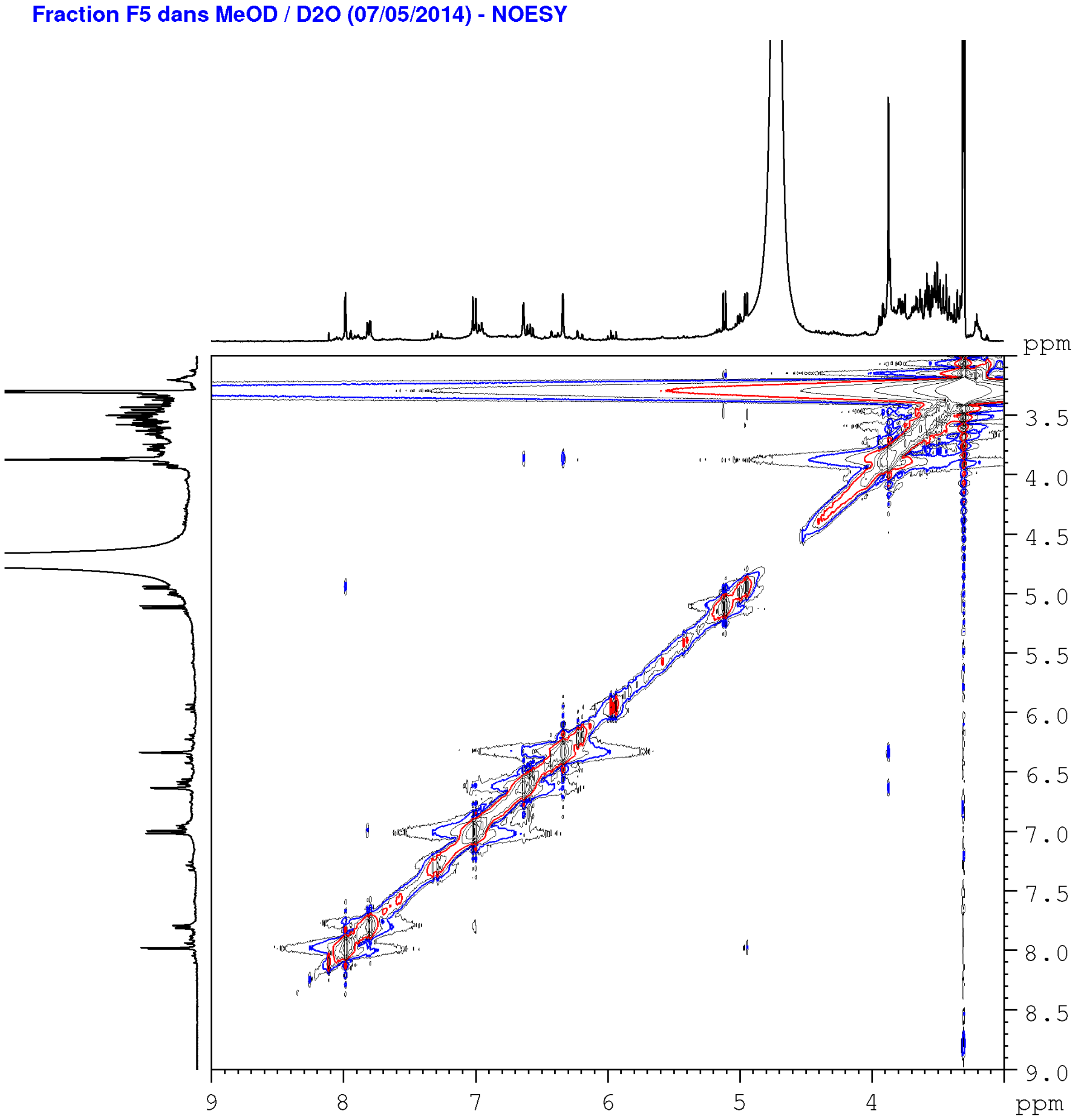

Supplement: Supplementary file 1 [file molecules-20-18128-s001.pdf]
